# Fluorescence Lifetime Imaging Unravels *C. trachomatis* Metabolism and Its Crosstalk with the Host Cell

**DOI:** 10.1371/journal.ppat.1002108

**Published:** 2011-07-14

**Authors:** Márta Szaszák, Philipp Steven, Kensuke Shima, Regina Orzekowsky-Schröder, Gereon Hüttmann, Inke R. König, Werner Solbach, Jan Rupp

**Affiliations:** 1 Institute of Medical Microbiology and Hygiene, University of Lübeck, Lübeck, Germany; 2 Department of Ophthalmology, UK-SH, Campus Lübeck, Lübeck, Germany; 3 Institute of Biomedical Optics, University of Lübeck, Lübeck, Germany; 4 Institute of Medical Biometry and Statistics, University of Lübeck, Lübeck, Germany; 5 Medical Clinic III, UK-SH/Campus Lübeck, Lübeck, Germany; Duke University, United States of America

## Abstract

*Chlamydia trachomatis* is an obligate intracellular bacterium that alternates between two metabolically different developmental forms. We performed fluorescence lifetime imaging (FLIM) of the metabolic coenzymes, reduced nicotinamide adenine dinucleotides [NAD(P)H], by two-photon microscopy for separate analysis of host and pathogen metabolism during intracellular chlamydial infections. NAD(P)H autofluorescence was detected inside the chlamydial inclusion and showed enhanced signal intensity on the inclusion membrane as demonstrated by the co-localization with the 14-3-3β host cell protein. An increase of the fluorescence lifetime of protein-bound NAD(P)H [τ_2_-NAD(P)H] inside the chlamydial inclusion strongly correlated with enhanced metabolic activity of chlamydial reticulate bodies during the mid-phase of infection. Inhibition of host cell metabolism that resulted in aberrant intracellular chlamydial inclusion morphology completely abrogated the τ_2_-NAD(P)H increase inside the chlamydial inclusion. τ_2_-NAD(P)H also decreased inside chlamydial inclusions when the cells were treated with IFNγ reflecting the reduced metabolism of persistent chlamydiae. Furthermore, a significant increase in τ_2_-NAD(P)H and a decrease in the relative amount of free NAD(P)H inside the host cell nucleus indicated cellular starvation during intracellular chlamydial infection. Using FLIM analysis by two-photon microscopy we could visualize for the first time metabolic pathogen-host interactions during intracellular *Chlamydia trachomatis* infections with high spatial and temporal resolution in living cells. Our findings suggest that intracellular chlamydial metabolism is directly linked to cellular NAD(P)H signaling pathways that are involved in host cell survival and longevity.

## Author Summary

Separate analysis of host and pathogen metabolic changes in intracellular *C. trachomatis* infections is arduous and has not been comprehensively realized so far. A more detailed understanding about the metabolic activity and needs of *C. trachomatis* and its specific interactions with the host cell would be the basis for the development of novel treatment strategies. We therefore applied fluorescence lifetime imaging (FLIM) of the metabolic coenzymes NAD(P)H using two-photon microscopy to directly visualize metabolic changes of host cells and pathogens in living cells. NAD(P)H fluorescence was detected both on the chlamydial inclusion membrane and inside the inclusion. Interestingly, changes in chlamydial growth and progeny induced by glucose starvation and IFNγ treatment were directly linked to significant changes of the NAD(P)H fluorescence lifetimes inside the inclusions. Furthermore, measurement of the NAD(P)H fluorescence lifetime in the host cell nucleus revealed that infected cells were programmed for starvation during the metabolically active phase of intracellular chlamydial growth. Our findings highlight for the first time a direct interaction between host and pathogen metabolism in intracellular bacterial infections that exceeds sole competition for nutrients. In conclusion, fluorescence lifetime imaging of NAD(P)H by two-photon microscopy enables real-time analysis of metabolic host-pathogen interactions in intracellular infections with high spatial and temporal resolution.

## Introduction

The obligate intracellular bacterium *Chlamydia trachomatis* (*C. trachomatis*) has two metabolically different developmental forms, which ensure its infectivity and replication. The infectious form, the elementary body, enters the host cell and differentiates into a metabolically active form, the reticulate body. Chlamydial reticulate bodies grow within the host cell in an intracellular membrane-bound compartment called the chlamydial inclusion. Within 24 hours post infection (hpi), numbers and size of reticulate bodies are maximized and chlamydiae start to re-differentiate to infectious elementary bodies. Cell lysis and release of the elementary bodies occur at around 48 hpi [1].

*C. trachomatis* is a sensitive marker organism for host cell metabolic changes because it strongly depends on ATP and metabolites generated by the host. Although an ADP/ATP transporter has been found [2], [3], the genome sequence also unraveled the existence of several glucose metabolizing enzymes [4]. Since then, it has been speculated that *C. trachomatis* not only uses host cell ATP, but also is capable to produce its own energy [5]. However, the metabolic pathways of *C. trachomatis* are often truncated. Thus, *C. trachomatis* might directly import the substrates required to compensate for the incomplete metabolic pathways [4]. Using microarray technology, it was shown that the ADP/ATP translocase and the ATP requiring oligopeptide transporters are expressed as immediate early genes. Furthermore, some metabolic enzymes such as the malate dehydrogenase (which requires nicotinamide-adenine dinucleotide (NAD) as cofactor) are also expressed in the early phase of infection [6]. Interestingly, no pathways for the biosynthesis of NAD and no NAD kinase for the synthesis of phosphorylated NAD have been found in the chlamydial genome. Although it seems obvious that a system to import NAD(P) from the host cell must exist similarly to that of environmental chlamydiae, no NAD(P) transporter has yet been identified in *C. trachomatis* according to sequence homology searches [7]. It is therefore reasonable to assume that intracellular chlamydial development strongly depends on host cell NAD availability.

The lack of suitable methods to investigate chlamydial metabolism separately from host cell metabolism has hindered scientific progress in studying host and pathogen metabolic interactions. Current knowledge on chlamydial metabolism is restricted to micro-array and RT-PCR analyses about the expression of metabolic genes during different intracellular developmental stages, the characterization of recombinant chlamydial metabolic enzymes, and the biochemical analysis of infected cells [1], [5], [6], [8]–[10]. Recently, it was shown by Raman microspectroscopy that amino acid uptake and protein synthesis is preserved in *C. trachomatis* after extracellular incubation [11]. Increases in glucose consumption and ATP levels were observed in cells infected with chlamydiae, however these studies could not distinguish between host cell and bacterial metabolism [12], [13].

Fluorescence lifetime imaging (FLIM) of NAD(P)H using two-photon laser scanning microscopy has been used to study cellular metabolism in living cells by utilizing the autofluorescence properties of the reduced form of the metabolic cofactor, NAD [NAD(H)] and its phosphorylated form, NADP [NAD(P)H] [14]–[17]. At 730–750 nm excitation, the cellular autofluorescence as measured by two-photon microscopy is dominated by NAD(P)H [14], [16], [18], [19]. The fluorescence lifetime is the time that a molecule spends to return to its ground state from its excited state. This exponential decay rate can be measured by time correlated single photon counting (TCSPC). The fluorescence lifetime of a molecule is thereby independent of its total concentration but strongly depends on its respective protein binding within a given microenvironment [20]. Thus, NAD(P)H functions are determined by binding to different cellular proteins. NAD(H) is a cofactor for catabolic reactions in the cytosol and the mitochondria while the phosphorylated NADP(H) is a cofactor in anabolic reactions and plays an important role in the cellular anti-oxidative defense system [21], [22]. NADH is generated within the glycolytic pathway by glyceraldehyde-phosphate-dehydrogenase (GAPDH) in the host cell cytosol while NADPH is produced by the enzymes of the pentose-phosphate pathway. In the mitochondria, NAD(P)H cannot pass through the inner mitochondrial membrane and only electrons from NAD(P)H are carried across by the malate-aspartate shuttle for oxidative phosphorylation. By contrast, free NAD(P)H can cross the nuclear envelope by diffusion through the nuclear pores; therefore, changes of cytosolic levels of free NAD(P)H are represented in the nucleus. An important binding protein of NADH in the nucleus is the transcriptional co-repressor, C-terminal binding protein (CTBP) [23]. Consequently, nuclear NADH functions to control gene transcription by regulating CTBP. [24]. Importantly, fluorescence lifetimes of NAD(P)H significantly differ between cytosol, mitochondria and nucleus in one single cell [19]. Differences of the NAD(P)H fluorescence lifetimes that are based on the different protein-bindings of NAD(P)H reflect the diverse physiological functions in these cellular compartments. Thus, monitoring of NAD(P)H fluorescence lifetimes allow for a compartmentalized analysis of cellular metabolic changes over time. In addition, calculating the bi-exponential fluorescence decay enables not only the monitoring of different components of the fluorescence lifetimes but also the ratio of free to protein-bound NAD(P)H [14], [15]. The fluorescence lifetime of protein-bound NAD(P)H [τ_2_-NAD(P)H] is longer than that of free NAD(P)H [τ_1_-NAD(P)H] [20]. As free NAD(P)H is readily diffusible, regulation of its binding proteins is correlated to the relative amount of free NAD(P)H [24]. The value of τ_2_-NAD(P)H and the ratio of free to protein-bound NAD(P)H are established markers to sensor metabolic activity of eukaryotic cells and have been used to monitor changes in host cell glycolysis or oxidative phosphorylation [15], [19], [25]. Inhibition of glycolysis results in an increase of τ_2_-NAD(P)H while the inhibition of oxidative phosphorylation decreases τ_2_-NAD(P)H. The shift from oxidative phosphorylation to glycolysis has been made responsible for the decrease of τ_2_-NAD(P)H during cancer development [15]. Furthermore, FLIM is the only currently available method to determine compartmentalized redox state of NAD(P)H in living cells.

We applied two-photon FLIM of NAD(P)H to discriminate and thoroughly characterize chlamydial and host cell metabolism. By measuring τ_2_-NAD(P)H values and determining the ratio of free to protein-bound NAD(P)H in *C.trachomatis*-infected cells, we observed significant changes both in host cell and chlamydial metabolism during the intracellular developmental cycle.

## Results

### Compartmentalization of NAD(P)H in *C. trachomatis*-infected cells

**Figure 1:**
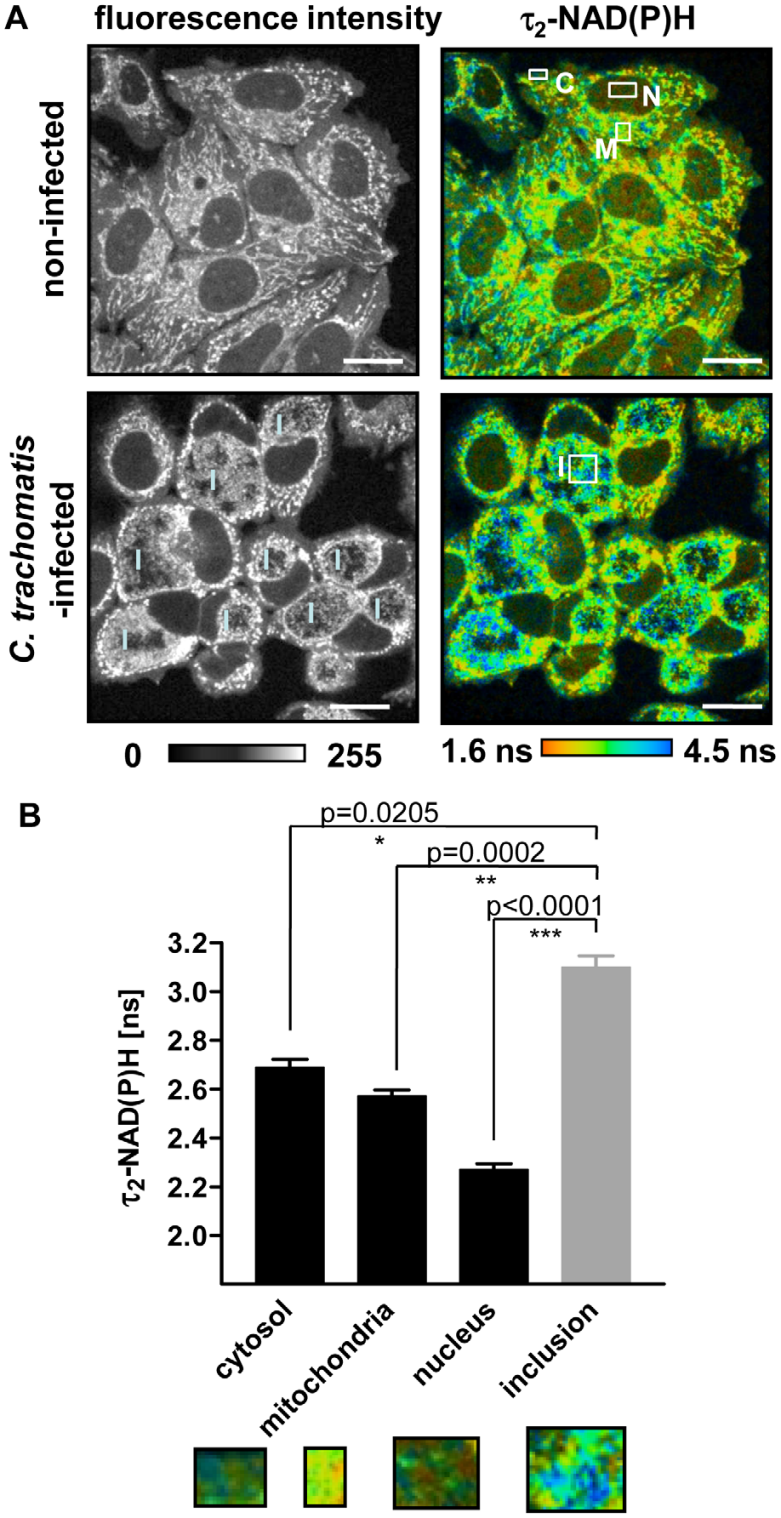
NAD(P)H fluorescence intensity signals and τ_2_-NAD(P)H in non-infected and *C.trachomatis*-infected cells. (A) FLIM of NAD(P)H by two-photon microscopy in non-infected (upper panels) and *C. trachomatis*-infected (lower panels) HEp-2 cells at 24 hpi. Left panels: Greyscale images of NAD(P)H fluorescence intensity signals. Right panels: Color-coded images of τ_2_-NAD(P)H. White squares show ROIs of cellular compartments used for quantitative analysis of τ_2_-NAD(P)H (C: cytosol, M: mitochondria, N: nucleus, I: inclusion; scale bar = 20 µm). (B) Quantitative analysis of τ_2_-NAD(P)H in the cytosolic, mitochondrial and nuclear compartments of non-infected cells and in the chlamydial inclusion of infected cells showed significantly increased τ_2_-NAD(P)H inside the chlamdial inclusions compared to other cellular compartments at 24 hpi. (n = 54 ROIs from three independent experiments, mean±SEM). Detailed results of statistical analysis are shown in Table S2. Images show enlargement of representative ROIs used for analysis. ROIs were selected by signal intensities. Low intensity areas were selected as cytoplasma and nucleus, high intensity areas as mitochondria. For the analysis of the inclusions, ROIs were selected inside the inclusion (see Figure S2A and S2B).

In non-infected HEp-2 cells, the majority of cellular autofluorescence at 730 nm excitation originated from the mitochondria. The cytosol and nuclei showed reduced fluorescence, representing the compartmentalized distribution of NAD(P)H in the cells (Figure 1A). To confirm that NAD(P)H is the major contributor to cellular autofluorescence at 730 nm excitation, the autofluorescence spectra were measured in four different emission channels. The highest emission intensity of autofluorescence was between 380 nm and 500 nm in all cellular compartments, corresponding to the peak of NAD(P)H fluorescence emission (Figures S1A and S1B). In *C. trachomatis*-infected cells a new compartment is formed, the chlamydial inclusion which also showed a strong autofluorescence signal (Figure 1A). The spectra of the chlamydial fluorescence did not show any shift compared to the autofluorescence of the other cellular compartments, indicating that the fluorescence inside the chlamydial inclusion also originated primarily from NAD(P)H (Figures S1A and S1B). The chlamydial inclusions could be clearly distinguished from the host cells due to the different fluorescence intensity of NAD(P)H inside the chlamydial inclusion (Figures S2A and S2B). The selected region of interest (ROI) was then used to determine fluorescence lifetimes of NAD(P)H inside the chlamydial inclusion and other cellular compartments. We observed a broad distribution of τ_2_-NAD(P)H that indicates heterogeneity of NAD(P)H protein binding in each cellular compartment (Figure S2C). We could confirm that the mean values of τ_2_-NAD(P)H in the cytosol, nucleus, mitochondria and chlamydial inclusion were significantly different (overall significance *p*<0.0001). The mean lifetime values are listed in Table S1. In addition, τ_2_-NAD(P)H was significantly increased inside the chlamydial inclusion at 24 hpi compared to all the other cellular compartments (Figure 1B).

### Non-mitochondrial NAD(P)H fluorescence surrounding the chlamydial inclusion

**Figure 2:**
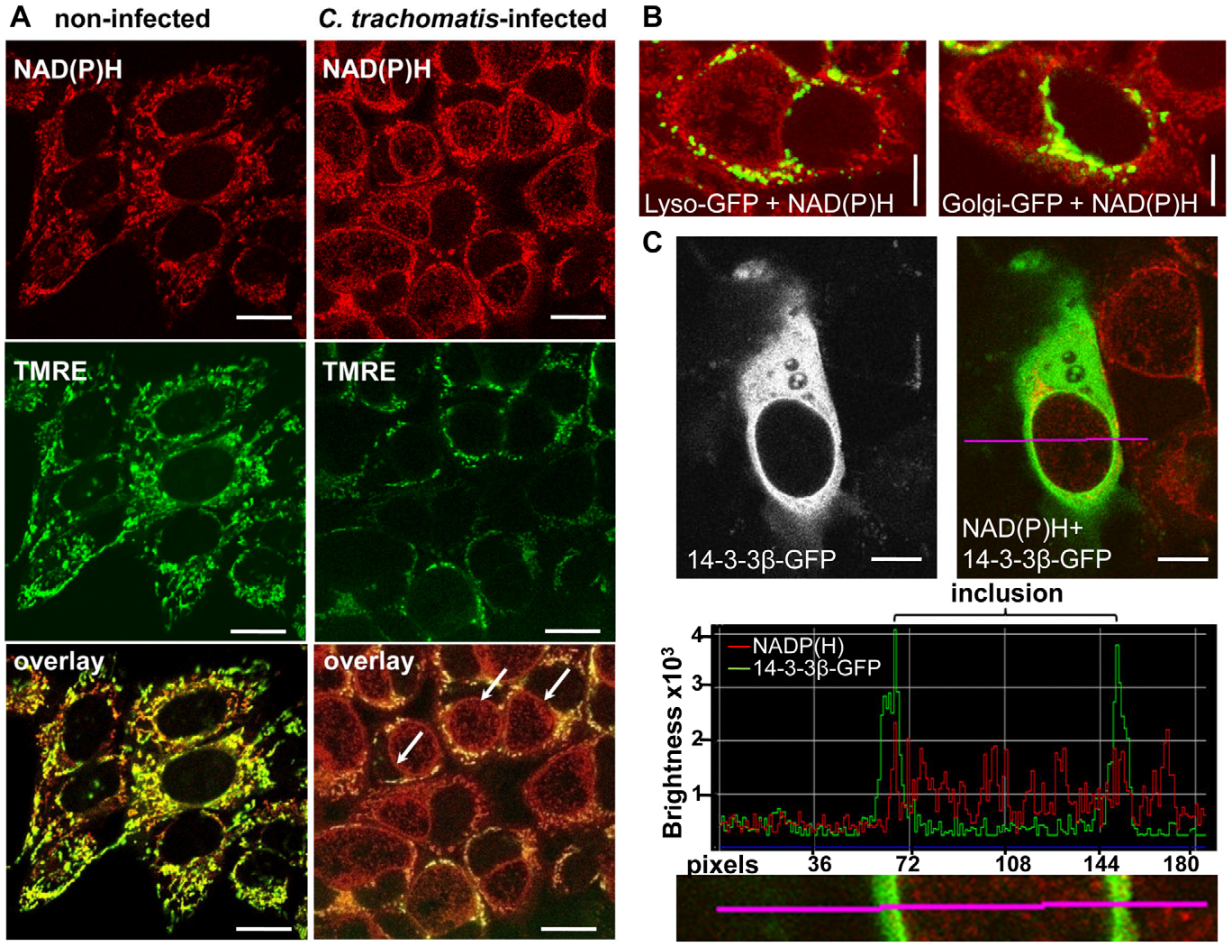
Non-mitochondrial NAD(P)H fluorescence in *C. trachomatis*-infected cells. (A) In non-infected HEp-2 cells (left panels), the mitochondria marker TMRE (middle panels; fluorescence emission between 580–680 nm, depicted in green) co-localizes (lower panels) with the high intensity NADP(H) fluorescence signal (upper panels; fluorescence emission between 450–500 nm, depicted in red). In *C. trachomatis*-infected cells (24 hpi, right panels), the arrows point to high intensity non-mitochondrial NAD(P)H signals of the chlamydial-inclusions. TMRE and NAD(P)H were excited at 730 nm (scale bar = 20 µm). (B) CellLight Lysosomes-GFP (left panel) and CellLight Golgi-GFP (right panel) did not co-localize with NAD(P)H autofluorescence in *C. trachomatis*-infected cells at 24 hpi. GFP was excited at 840 nm and NAD(P)H was excited at 730 nm. Pictures are overlays of pseudocolor images (GFP = green, NAD(P)H = red; scale bar = 10 µm). (C) Overexpressed 14-3-3β-GFP protein co-localized with the NAD(P)H autofluorescence in *C. trachomatis*-infected cells at 24 hpi. 14-3-3β-GFP showed increased fluorescence at the chlamydial inclusion membrane (left panels). Pseudocolor image (right panel) of NAD(P)H fluorescence (excited at 730 nm, red) and GFP fluorescence (excited at 840 nm, green) shows the overlay. The fluorescence intensity profile (middle panel) of the line indicated in the original and in the magnified picture (lower panel) confirm the co-localization of 14-3-3β-GFP and NAD(P)H fluorescence at the chlamydial inclusion membrane (scale bar = 10 µm).

NAD(P)H fluorescence showed an increased intensity surrounding the chlamydial inclusion. To test whether the high intensity signal originated from the host cell mitochondria, we co-incubated the cells with a mitochondria marker, tetramethylrhodamine-ethyl-ester (TMRE). TMRE showed complete co-localization with the high intensity NAD(P)H fluorescence in non-infected HEp-2 cells (Figures 2A and S3A). By contrast, in *C. trachomatis*-infected cells, high intensity signals only partially co-localized with TMRE. Most of the high intensity fluorescence which was found around the chlamydial inclusion did not co-localize with host cell mitochondria that were labeled with TMRE (Figures 2A and S3B). This high intensity signal around the inclusion also did not show co-localization with the GFP-tagged lysosomal and golgi markers that were overexpressed in *C. trachomatis*-infected cells (Figure 2B). Overexpression of the GFP-tagged 14-3-3β protein that was previously shown to be localized on the inclusion membrane [26] co-localized with NAD(P)H indicating that the high intensity NAD(P)H fluorescence derives from the inclusion membrane in *C. trachomatis*-infected cells (Figure 2C).

### Fluorescence lifetime changes of NAD(P)H in the chlamydial inclusions during the developmental cycle

**Figure 3:**
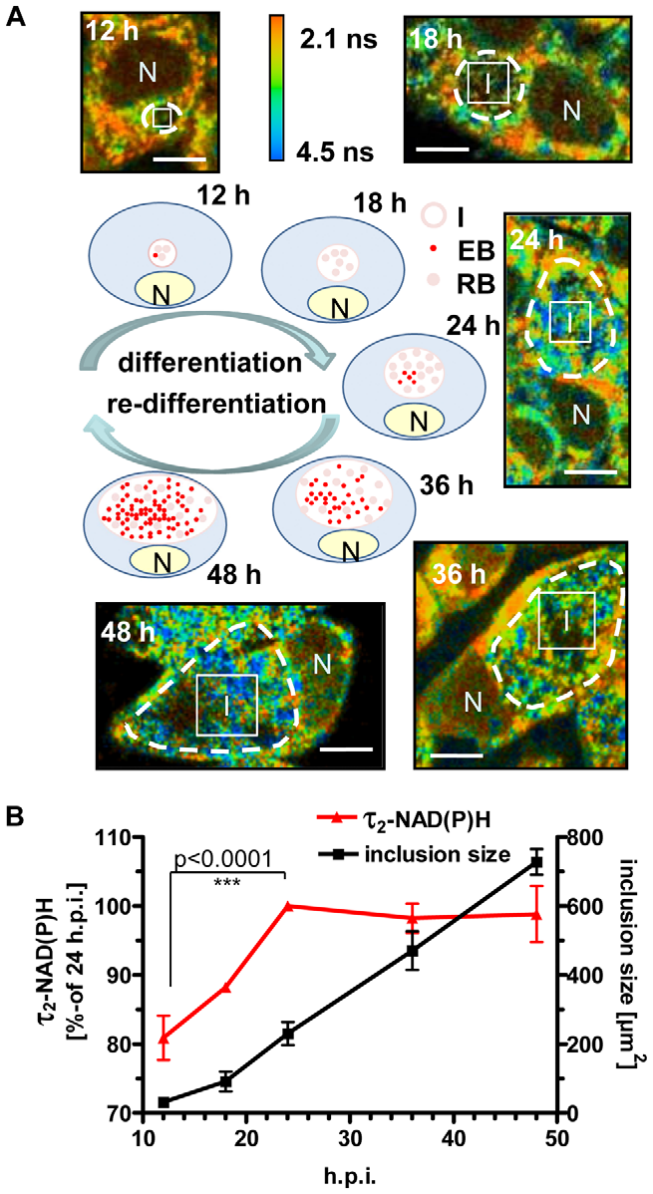
Changes of τ_2_-NAD(P)H inside the chlamydial inclusions during the intracellular developmental cycle. HEp-2 cells were infected with *C. trachomatis,* for 12, 18, 24, 36 and 48 hours. (A) Schematic presentation of bacterial inclusion formation in *C. trachomatis*-infected cells (EB = elementary body, RB = reticulate body, N = nucleus, I = inclusion). Representative color-coded images of τ_2_-NAD(P)H in *C. trachomatis*-infected cells at the indicated times of infection are shown (scale bar = 10 µm). White squares show ROIs inside the chlamydial inclusion used for quantitative analysis of τ_2_-NAD(P)H. Chlamydial inclusions are marked by dashed lines. (B) Quantitative analysis of τ_2_-NAD(P)H inside the chlamydial inclusion and of chlamydial inclusion sizes (n = 54 from three independent experiments; mean±SEM). Detailed results of statistical analysis are shown in Table S3A.

To verify our hypothesis that changes in the values of τ_2_-NAD(P)H inside the chlamydial inclusion are an indicator of chlamydial metabolism, we followed the alterations of τ_2_-NAD(P)H during the developmental cycle of *C. trachomatis* (Figure 3A). Increased metabolism of reticulate bodies during the mid-phase of intracellular growth was directly correlated with an increase of τ_2_-NAD(P)H between 12 and 4 hpi. By contrast, τ_2_-NAD(P)H did not change further during the late phase of the infection (48 hpi) when infectious elementary bodies were formed (Figure 3B). However, the distribution of τ_2_-NAD(P)H was more heterogeneous at 48 hpi (Figure S4A) whereas the ratio of free to protein-bound NAD(P)H (a_1_/a_2_ ratio) inside the chlamydial inclusion significantly decreased between 12 and 24 hpi (Figure S4B).The distribution and intensity of fluorescence in the chlamydial inclusions also changed during the developmental cycle. While highest fluorescence in the mid-phase of the chlamydial development was observed at the inner border of the inclusion, the average fluorescence intensity increased and was homogeneously distributed within the inclusion within 24 hpi. During late infection, large areas inside the inclusion showed no NAD(P)H fluorescence, indicating replacement of metabolically active reticulate bodies by infectious but metabolically inert elementary bodies (Figures S5A, S5B and S5C). An increase of τ_2_-NAD(P)H was also observed in inclusions from early *C. pneumoniae* infected cells, followed by a decline in τ_2_-NAD(P)H during later time points of the infection (Figures S6A and S6B).

### Inhibition of host cell metabolism directly interferes with the development and metabolism of *C. trachomatis*

**Figure 4:**
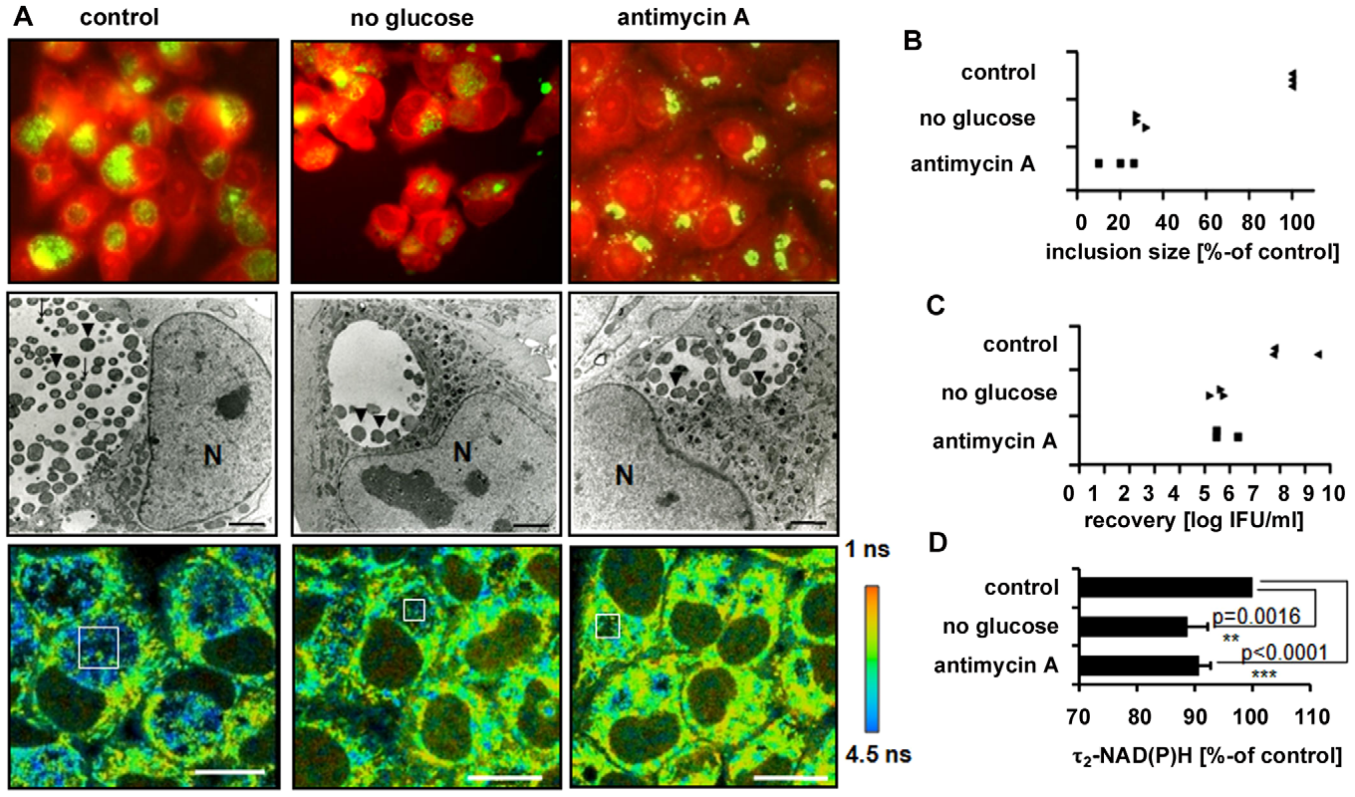
Impact of host-cell metabolism inhibition on chlamydial development and τ_2_-NAD(P)H inside chlamydial inclusions. HEp-2 cells, infected with *C. trachomatis,* were left untreated (control) or were grown in media without glucose or were treated with 3 nM antimycin A for 24 hours. (A) Upper panels: Immunofluorescence staining images show chlamydial inclusions in green (chlamydial-LPS antibody) and host cells in red (Evans-blue). Middle panels: Electron microscopic pictures show morphologically aberrant chlamydial development under glucose starvation and antimycin A treatment at 24 hpi. Arrows indicate EBs while arrowheads indicate RBs inside the chlamydial inclusions (N = nucleus; scale bar = 2 µm). Lower panels: Color-coded images of τ_2_-NAD(P)H. White squares inside the chlamydial inclusions show representative ROIs used for quantitative analysis (scale bar = 20 µm). (B) Inhibition of host cell metabolism decreases the sizes of chlamydial inclusions. Quantitative analyzes of chlamydial inclusion sizes at 24 hpi. Average sizes of 54 inclusions from three independent experiments are shown as percentage of control. (C) Inhibition of host cell metabolism decreases the number of infectious chlamydial progeny. Quantitative analysis of *C. trachomatis* recovery rates at 24 hpi. Averages of duplicates from three independent experiments are shown. (D) Inhibition of host cell metabolism decreases τ_2_-NAD(P)H inside the chlamydial inclusion. Quantitative analysis of τ_2_-NAD(P)H (n = 54 ROIs from three independent experiments; mean±SEM) at 24 hpi. Results are shown as percentage of control. Detailed results of statistical analysis are shown in Table S4.

To further prove that τ_2_-NAD(P)H characterizes the metabolic activity of *C. trachomatis*, we monitored τ_2_-NAD(P)H changes inside the chlamydial inclusion during inhibition of host cell metabolism. We used glucose starvation to inhibit host cell glycolysis and antimycin A to inhibit the mitochondrial complex III and subsequent oxidative phosphorylation. Both inhibitors decreased host cell ATP levels (Figure S7A). The intracellular development of *C. trachomatis* was monitored by immunofluorescence staining and electron microscopic (EM) imaging of chlamydial inclusions (Figure 4A). Sizes of chlamydial inclusions were quantitatively analyzed at 24 hpi and the numbers of infectious *C. trachomatis* progeny were measured by recovery assays (Figures 4B and 4C). Both glucose starvation and treatment with antimycin A resulted in smaller and aberrant chlamydial inclusions when compared to untreated cells. Quantification of the fluorescence lifetimes showed a significant decrease of τ_2_-NAD(P)H inside the *C. trachomatis* inclusions when the host cells were treated with the metabolic inhibitors, as an indicator for decreased metabolism of *C. trachomatis* (Figures 4D and S7B). The observed decrease in the τ_2_-NAD(P)H values was directly correlated to the decreased recovery rate of infectious *C. trachomatis*.

### Decreased τ_2_-NAD(P)H and relative amount of protein-bound NAD(P)H in persistent chlamydial inclusions

**Figure 5:**
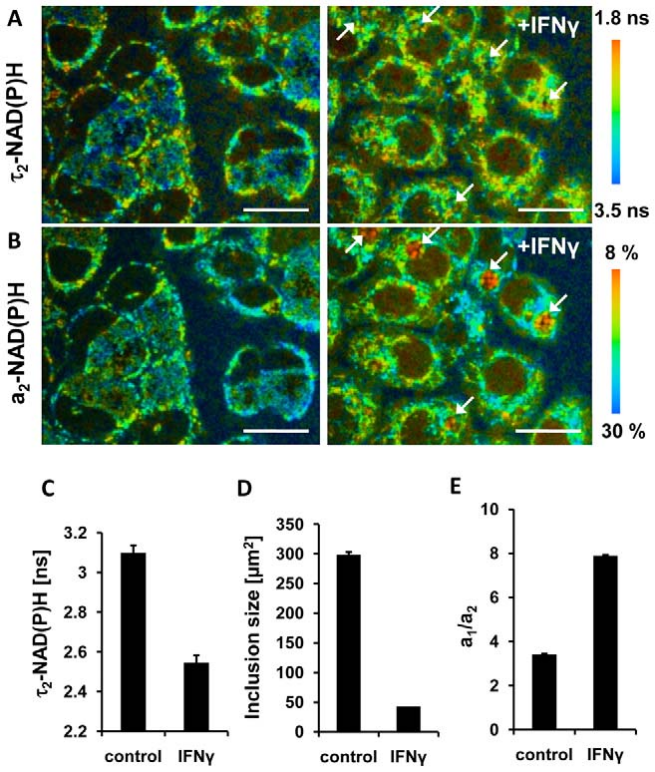
Effects of IFNγ treatment on NAD(P)H inside chlamydial inclusions. HEp-2 cells were infected with *C. trachomatis* for 24 hours and treated with 10 units /ml IFNγ 24 hours prior to and during the infection (right panels) or were left untreated (left panels). (A) Color-coded images of τ_2_-NAD(P)H (scale bar = 20 µm). Arrows point to persistent chlamydial inclusions harbouring enlarged reticulate bodies. (B) Color-coded images of the relative amount of protein-bound a_2_-NAD(P)H (scale bar = 20 µm). Arrows point to persistent chlamydial inclusions harbouring enlarged reticulate bodies. (C) IFNγ induced persistence decreases τ_2_-NAD(P)H inside the chlamydial inclusion. Quantitative analysis of τ_2_-NAD(P)H (n = 27 ROIs from three independent experiments; mean ±SEM) at 24 hpi. (D) IFNγ induced persistence decreases the sizes of chlamydial inclusions. Quantitative analyzes of chlamydial inclusion sizes at 24 hpi (n = 27 inclusions from three independent experiments; mean ±SEM). (E) IFNγ induced persistence increases the ratio of a_1_/a_2_ inside the chlamydial inclusions. Quantitative analysis of ratio of free to protein-bound NAD(P)H (a_1_/a_2_) (n = 27 ROIs from three independent experiments; mean ±SEM).

Interferon-γ (IFNγ) is known to induce *C. trachomatis* persistence that is defined as a viable but non-cultivable developmental stage. Persistent *C. trachomatis* are characterized by enlarged reticulate bodies in morphological aberrant inclusions [27]. To determine the metabolism of reticulate bodies in persistence, we measured the τ_2_-NAD(P)H values inside the chlamydial inclusions in IFNγ-treated cells 24 hpi. The mean values of τ_2_-NAD(P)H (Figures 5A and 5C) and the size of the inclusions (Figure 5D) were decreased in persistent infections. The relative amount of protein-bound a_2_-NAD(P)H was also largely reduced resulting in an increase of the a_1_/a_2_ ratio (Figures 5B and 5E).

### Decrease in the relative amount of free NAD(P)H in the host cell nucleus indicates cellular starvation in *C. trachomatis*-infected cells

**Figure 6:**
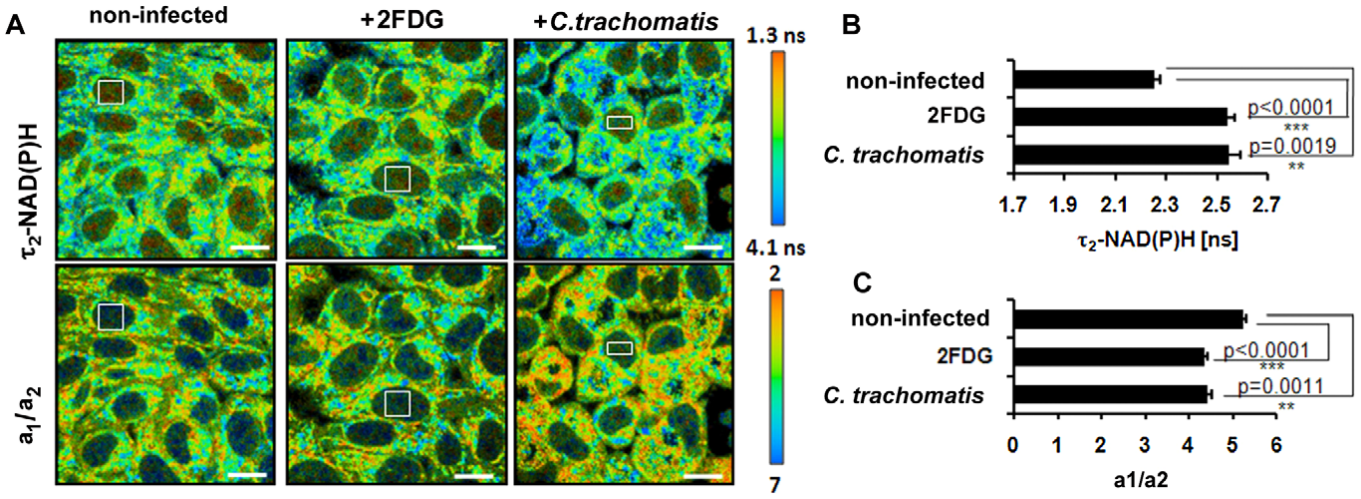
Cellular starvation in *C. trachomatis*-infected cells. HEp-2 cells were left untreated (control) or were treated with 5 mM 2FDG or were infected with *C. trachomatis* for 24 hours. (A) Upper panels: Color-coded images of τ_2_-NAD(P)H. Lower panels: Color-coded images of the a_1_/a_2_ ratio. White squares inside the nucleus show representative ROIs used for quantitative analysis (scale bar = 20 µm). (B) Treatment of cells with 2FDG or infection with *C. trachomatis* increases τ_2_-NAD(P)H inside the nucleus. Quantitative analysis of τ_2_-NAD(P)H (n = 54 ROIs from three independent experiments; mean±SEM). Detailed results of statistical analysis are shown in Table S5A. (C) Treatment of cells with 2FDG or infection with *C. trachomatis* decreases the a_1_/a_2_ ratio inside the nucleus. Quantitative analysis of a_1_/a_2_ ratio (n = 54 ROIs from three independent experiments; mean ±SEM). Detailed results of statistical analysis are shown in Table S5B.

In the late phase of *C. trachomatis*-infection, FLIM analysis of cellular NAD(P)H was impeded by the large sizes of the chlamydial inclusions filling out almost the whole cytosol of the cells. As free NAD(P)H is diffusible, cytosolic levels are reflected in the cell nucleus. To characterize host cell metabolism during later stages of the infection, we measured the ratio of free to protein-bound NAD(P)H (a_1_/a_2_) and the values of τ_2_-NAD(P)H in the nucleus. The ratio of a_1_/a_2_ was decreased and τ_2_-NAD(P)H was increased in the nucleus of *C. trachomatis*-infected cells at 24 hpi (Figure 6A-C). Similar effects were observed when host cell glycolysis was inhibited by 2-fluoro-deoxy-glucose (2FDG) or by glucose deprivation (data not shown) that mimic cellular starvation. The results indicate decreased levels and altered protein binding of NAD(P)H in the nucleus as a consequence of metabolic changes in the host cell cytosol.

## Discussion

*C. trachomatis* was speculated to be an energy parasite that totally depends on the supply of energy and metabolic co-enzymes from the nutrient-rich cytoplasm of an infected host cell. In recent years, when genome data became available and with the use of functional enzymatic competition assays, it became obvious that chlamydiae contain the capacity to produce its own energy and reducing power [5]. However, separate analysis of host and pathogen metabolism is still challenging and requires novel experimental and technical set-ups that facilitate dynamic monitoring of metabolic changes inside the intracellular chlamydial inclusion separately from the host cell in living cells. Imaging of NAD(P)H by its autofluorescence is an ideal tool to study metabolism in *C. trachomatis*-infected cells as it permits the spatial separation of metabolic changes in different cellular compartments [19]. By use of the two-photon microscopy technique lateral resolutions of below 1 µm are achieved with a maximum reduction in photo-damage over time. Changes in the NAD(P)H fluorescence lifetimes thereby reflect different protein binding characteristics of NAD(P)H and allow to calculate the relative amount of free NAD(P)H in ratio to protein-bound NAD(P)H [14].

First we studied the compartmentalized distribution of NAD(P)H fluorescence in *C. trachomatis*-infected cells. This uncovered a high intensity NAD(P)H fluorescence around the chlamydial inclusion which was not of mitochondrial origin. Previous findings that the *C. trachomatis*-inclusion does not co-localize with the mitochondria of the host cells were confirmed [28]. Unlike the nuclear pore, the chlamydial inclusion is passively impermeable to small molecules rendering sole diffusion of NAD(P)H across the inclusion membrane unlikely [29]. The co-localization of the non-mitochondrial NAD(P)H signal with the inclusion marker 14-3-3β host cell protein indicates that it might represent a potential NAD(P)H transporter or the active site where NAD(P)H is synthesized for chlamydial use. The existence of NAD(P) transporter in environmental *chlamydiae* [7] suggests that human pathogenic *chlamydiae* also harbour a transporter rather than a biosynthetic pathway because of its smaller genome size.

Our data on the distribution of NAD(P)H fluorescence within the chlamydial inclusion further supports the model from Wilson *et al.* that reticulate bodies first occupy the juxta-membrane space during the formation of the chlamydial inclusion and later move to the centre for differentiation [30]. In the centre of the inclusion, limited supply of essential metabolites may ultimately trigger their re-differentiation to infectious elementary bodies [31].

The significantly increased τ_2_-NAD(P)H values inside the chlamydial inclusion define a novel, metabolically distinct cellular compartment within infected cells. Comparison of the classical NAD(P) binding domains in humans and *C. trachomatis* using the SUPERFAMILY database search (http://supfam.cs.bris.ac.uk/SUPERFAMILY_1.73/) [32] indicated that the Rossmann-fold [33] is present in 318 proteins in humans but only in 14 proteins of *C. trachomatis* (L2b/UCH-1/proctitis). Our measurements of τ_2_-NAD(P)H values during the developmental cycle indicate that NAD(P)H within *C. trachomatis* inclusion binds to different proteins as its metabolism changes. In addition, our results indicate that τ_2_-NAD(P)H strongly correlates with the metabolic activity of chlamydial reticulate bodies but not with the inclusion size. The increase in τ_2_-NAD(P)H in the mid- phase of infection was accompanied by a decrease in the relative amount of free NAD(P)H, which might indicate the presence of an oxidative type of energy metabolism within the chlamydial inclusion [4]. Similar changes of τ_2_-NAD(P)H were measured in *C. pneumoniae* inclusions. The longer developmental cycle of the bacteria allowed also monitoring the decrease in metabolism towards the end of the infection cycle. However the absolute changes that we could measure were also less pronounced.

IFNγ induces persistence of *C. trachomatis* [34]. The molecular mechanism involves tryptophan depletion through the induction of the indoleamine 2,3-dioxygenase (IDO) by IFNγ [35]. By measuring τ_2_-NADP(H) and the ratio of a_1_/a_2_ in the persistent chlamydial inclusions we could prove that the metabolism of the chlamydial reticulate bodies are largely reduced. In addition, we could visualize the enlarged reticulate bodies inside the inclusions.

Reduction of host cell ATP synthesis by inhibition of glycolysis or oxidative phosphorylation induced aberrant chlamydial inclusions and decreased τ_2_-NAD(P)H values in the chlamydial inclusions. Therefore, τ_2_-NAD(P)H values predicted metabolic activity of growing and differentiating reticulate bodies that resulted in infectious chlamydial progeny.

Looking more in detail on the ratio of free to protein-bound NAD(P)H in the host cell nucleus, we could also show that intracellular chlamydial infection programmed the cell for starvation. Free NAD(P)H, which is approximately 80% of the total amount of NAD(P)H, represents the freely diffusing NAD(P)H molecules as described by measuring fluorescence lifetime of NADH in solution [36]. As there is no barrier for the diffusion of free NAD(P)H between the cytosolic and nuclear compartments, nuclear NAD(P)H concentrations directly reflect the free cytosolic NAD(P)H concentrations [24]. The τ_2_-NAD(P)H changes in the nuclei of *C. trachomatis*-infected cells were consistent with those found in 2FDG treated cells in which cellular glycolytic flux is inhibited and cellular starvation is induced within 24 hours [19], [24]. It is well accepted that calorie restriction and cellular starvation go along with the induction of gene expression patterns that allow energy conservation and directly regulate the lifespan of cells [37]. The deacetylase sirtuin1 (SIRT1) is a master regulator of longevity in the host cell nucleus and is directly regulated by its substrate NAD [38] while its transcription is regulated by the NADH-binding transcriptional corepressor, carboxy-terminal binding protein 1 (CTBP) [23], [24], [39].

A limitation of NADP(H) autofluorescence imaging is that conversion of NAD(P)H fluorescence intensity values to absolute concentration values is not straightforward because the different quantum yields of free and protein-bound NAD(P)H have to be calculated [40], [41]. In addition, the fluorescence decay parameters of the phosphorylated and non-phosphorylated forms of reduced NAD are the same and are indistinguishable. Although estimations of cellular concentrations suggest that a substantial part of the cellular fluorescence originates from NADPH rather than from NADH [21], we and others have demonstrated significant NAD(P)H fluorescence lifetime changes by inhibiting glucose metabolism corresponding to projected changes of cellular NADH concentration [15], [40], [42]. We would also expect that NAD(P) transport or synthesis is initiated very early in the chlamydial developmental cycle as gene-expression analysis indicates that metabolic enzymes requiring NAD as cofactor are expressed as immediate early genes [6]. However, because of the small size of the intracellular *C. trachomatis* inclusions in the first 12 hours of infection, a reliable analysis of the NAD(P)H fluorescence in the early phase of infection could not be performed.

A more detailed understanding about the metabolic activity and needs of *C. trachomatis* during the intracellular growth phase is needed to conceive novel therapeutic strategies to target the pathogen in its intracellular growth phase without affecting the host. Fluorescence lifetime imaging using two-photon microscopy bares new insights into the crosstalk between host and pathogen metabolism and suggests *C. trachomatis*-induced changes in sub-cellular NAD(P)H contents to directly interfere with nuclear NAD(P)H signaling pathways that are involved in cellular survival and longevity. In the process of understanding on how intracellular pathogens interfere with host cell metabolism, metabolic profiling of infected cells by FLIM of NAD(P)H will be an invaluable tool that complements established large scale genomic and proteomic approaches.

## Materials and Methods

### Cell culture and propagation of *C. trachomatis* and *C. pneumoniae*

HEp-2 epithelial cells (ATCC CCL-23) were cultured in Dulbecco's modified Eagle's Medium (DMEM) with glucose (4.5 g/l) (PAA,), or for glucose starvation studies, in glucose free DMEM (PAA, Germany) supplemented with 10% heat-inactivated fetal bovine serum (FBS), 4 mM L-glutamine, 110 mg/l sodium-pyruvate, 10 mg/ml gentamicin and 30 mM Hepes. For two-photon microscopy, 5×10^5^ HEp-2 cells per dish were cultured in 50 mm culture dishes. For recovery assays, 3×10^5^ HEp-2 cells per well were cultured in 6-well culture plates. Cells were allowed to adhere for 24 hours prior to infection with *C. trachomatis or C. pneumoniae.* Cells were grown at 37°C, in 5% CO_2_ humidified air. *C. trachomatis* strain L2 (ATCC VR-902B) and *C. pneumoniae* CWL029 strain (ATCC VR-1310) were purified on discontinuous density gradients. HEp-2 cells were infected with 1 inclusion forming unit (IFU) *C. trachomatis* per cell. HEp-2 cells were infected with 4 IFU *C. pneumoniae* per cell in the presence of 1 µg/ml cycloheximide by using centrifugation.

### FLIM of NAD(P)H by two-photon microscopy

For two-photon microscopic studies, HEp-2 cells were grown on cover glass in 50 mm culture dishes and infected with *C. trachomatis* or *C. pneumoniae* as described above. Cover glasses were examined in a MiniCeM chamber for microscopy (JenLab, Jena, Germany) fitted to a heated stage, which enabled live cell imaging. The two-photon microscope (DermaInspect; Jenlab) was equipped with a Chroma 640DCSPXR dichroic mirror (AHF analysentechnik AG, Tübingen, Germany) and a 40×/1.3 Plan-Apochromat oil-immersion objective (Zeiss, Göttingen, Germany). A tunable infrared titanium-sapphire femtosecond-laser (710–920 nm tuning range; MaiTai; Spectra Physics, Darmstadt, Germany) was used as an excitation source at 730 nm excitation for FLIM of NAD(P)H. Residual excitation light was blocked from the FLIM detector by a blue emission filter (BG39, Schott AG, Mainz, Germany). FLIM data were collected by a time-correlated single-photon counting (TCSPC) system (PMH-100-0, SPC-830, Becker & Hickl, Berlin, Germany). Single photon counting was done for 49.7 seconds per image. Fluorescence lifetimes were analyzed using the SPCImage software version: 2.9.5.2996 (Becker & Hickl). The average power for the cell imaging experiments was 12 mW, and the scan area for each image plane was 110×110 µm^2^ corresponding to 256×256 pixels. Photon count rates at the beginning and end of image acquisition were monitored to ensure that photo-bleaching did not occur. For the image analysis, the ROI inside the chlamydial inclusion was selected. The ROIs contained 42 to 1147 pixels depending on the chlamydial inclusion size. The lifetimes of 5×5 pixels in the ROI were averaged before. The lifetime decay curves were fit to a double exponential decay model, in which the fast decaying component corresponds to free NAD(P)H [τ_1_-NAD(P)H] and the slow decaying component corresponds to protein-bound NAD(P)H [τ_2_-NAD(P)H[ [14]. The instrument response function (IRF) was measured from the second harmonic generation signal of beta-barium-borate crystal and it was used in the lifetime fit model. The mean values of τ_2_-NAD(P)H of all pixels inside the ROIs were calculated. ROIs from three cells per microscopy field were analyzed from six different microscopy fields per chamber on three independent measurement days [n = 54 (3×6×3)]. To compare τ_2_-NAD(P)H of different infection times and metabolic states, values were normalized to the average τ_2_-NAD(P)H 24 hpi (100%) for each experimental day. For fluorescence intensity analysis of FLIM pictures, photon counts values of selected ROIs inside the chlamydial inclusion were exported to Excel from SPCImage analysis software. Values of each pixel inside the ROIs from three different cells were used to create a histogram of fluorescence intensity distribution.

### Detection of spectrally distributed autofluorescence and fluorescent labeling of mitochondria

Fluorescence light was collected in four emission channels simultaneously using a set of dichroic mirrors and four photomultiplier tubes (Hamamatsu R1294A and R1295A). Fluorescence emission was spectrally distributed as follows: channel 1: 380–450 nm, channel 2: 450–500 nm, channel 3: 500–580 nm, channel 4: 580–680 nm. Residual excitation light was blocked by a two-photon emission filter (E680SP, Chroma Technology Corp., Bellows Falls, VT). Fluorescence intensities in each emission channels were analyzed in medium and in ROIs in host cell mitochondria and chlamydial inclusion by ImageJ. Background fluorescence intensity was recorded in a dark measurement without excitation light and was subtracted from the autofluorescence intensity values. The peak of emission in channel 2 (emission between 450–500 nm) at 730 nm excitation by two-photon microscopy corresponds to the peak of NAD(P)H fluorescence spectrum [18], [43]. For the fluorescent detection of mitochondria, cells were pre-incubated with the mitochondrial membrane potential sensitive dye, tetramethylrhodamine-ethyl-ester (TMRE) (10 nM) for 15 minutes. Because of the overlap of the two-photon excitation cross-section between TMRE and NAD(P)H, simultaneous excitation of both fluorescence signals was possible at 730 nm. Fluorescence of channel 2 (NAD(P)H) and fluorescence of channel 4 (TMRE) were used for creating merged images by ImageJ.

### BacMam transduction, transfection

HEp-2 cells were transduced with CellLight Golgi-GFP BacMam 2.0 or CellLight Lysosomes-GFP BacMam 2.0 (Invitrogen). Transduction was performed simultaneously with *C. trachomatis* infection using 10 virus particles per cell. The plasmid encoding the carboxy terminally GFP-tagged 14-3-3β protein (RG215940) was purchased from Origene (Bethesda, MD). Transfection was performed 24 hours before *C. trachomatis* infection using 2 µl/ ml Fugene6 transfection reagent (Roche) and 1 µg / ml DNA according to the manufacturer's instruction.

### GFP Imaging and co-localization analysis

GFP was excited at 840 nm by the two-photon microscope described above. Cellular autofluorescence was minimal at 840 nm when low excitation power was used. Two-photon excitation of GFP fluorescence at 730 nm provided minimal absorption when GFP overexpression was at a low level. To analyze co-localization, intensity images were exported to the Keyence BZ9000 Analysis software (Osaka, Japan) and line intensity profiles of the fluorescence were calculated.

### Chlamydial inclusion size measurements

Chlamydial inclusion sizes were analyzed by measuring the area of inclusions after exporting the grey-scale FLIM intensity images from SPCImage software to ImageJ (NIH, Bethesda, MD). 54 inclusions were analyzed from three independent experiments.

### IFNγ induced persistence

To induce persistence, HEp-2 cells were treated with 10 units/ml IFNγ for 24 hours prior to and during *C. trachomatis* infection.

### Chlamydial recovery

To determine the burden of infectious *C. trachomatis* elementary bodies after intracellular development, titration experiments were performed. Infected HEp-2 cells (24 hpi) were mechanically detached with a cell scraper and re-suspended in fresh growth medium. Serial dilutions of suspension were inoculated in confluent cycloheximide-treated HEp-2 cell monolayers with centrifugation-assisted inoculation. Development of chlamydial inclusions after 30 hours was analyzed on methanol-fixed slides using FITC-labeled monoclonal chlamydial-LPS antibodies (Dako, Glostrup, Denmark). Chlamydial recovery was calculated as IFU/µl by observation of 10 microscopy fields (40×magnification).

### Metabolic inhibition of HEp-2 cells

To inhibit host cell glycolysis, glucose starvation or 2FDG (Sigma) treatment were used. To inhibit host cell oxidative phosphorylation, antimycin A (Sigma) treatment was used. HEp-2 cells were allowed to adhere for 24 hours. For glucose starvation, media was changed to media containing no glucose. For 2FDG and antimycin A treatment, media was changed to media containing 5 mM 2FDG or 3 nM antimycin A respectively. 15 minutes after the media was changed, cells were infected with *C. trachomatis* or were left un-infected. After 24 hours of infections, FLIM measurements, recovery assays, ATP measurements and immunofluorescence stainings were performed.

### Immunofluorescence staining

Growth media was changed to media containing no glucose or containing antimycin A (3 nM) prior to infection with *C. trachomatis*. After 24 hours of infection, cells were fixed with methanol and chlamydial inclusions were stained with FITC-labelled monoclonal chlamydial-LPS antibodies (Dako). For better visualization cells were counterstained with 0.1% Evans-blue.

### ATP measurements

ATP was measured using a luminometric ATP assay kit (ABD Bioquest, Sunnyvale, CA) and a microplate reader (Tecan Infinite 200 PRO, Maenedorf, Switzerland). HEp-2 cells were grown in 96-well plates (2000 cells/ well) and treated with the metabolic inhibitors as described above. ATP assays were performed according to the manufacturer's instructions.

### Electron microscopy

Cells were fixed with 2% paraformaldehyde and 2.5% glutaraldehyde in 0.1 M cacodylate buffer for 1 hour. Post-fixation was performed with 1% OsO4 in O.1 M cacodylate buffer for 2 hours. Samples were dehydrated with graded ethanol series and embedded in araldite (Fluka, Buchs, Switzerland). Ultrathin sections were stained with uranyl acetate and lead citrate and were examined with a Philips EM 400 transmission electron microscope.

### Statistical hypothesis testing

For all comparisons, we used nonparametric models for longitudinal data as described previously [44]. Due to small sample sizes, ANOVA-type statistics were applied with a Box-approximation. Adjustment for multiple testing: Overall, six sets of statistical tests were carried out (Figures 1B, 3B, 4D, 6B, 6C, S4B). The overall significance level was set to 0.05. Within each set, adjustment for multiple testing was performed using the hierarchical procedure by Bonferroni-Holm (Bonf-Holm). To account for the testing of six sets of hypotheses, a Bonferroni correction was performed overall.

### Accession numbers of proteins (Swiss-Prot):

1433B_HUMAN (YWHAB): P31946.

## Supporting Information

Figure S1 **Spectral characterization of autofluorescence signals at 730 nm excitation.** (A) Spectral characterization of autofluorescence signals of non-infected HEp-2 cells (upper panels) and of *C. trachomatis*-infected cells at 24 hpi (lower panels) shows typical NAD(P)H fluorescence (scale bar = 20 µm). (B) Quantification of fluorescence intensity of selected ROIs (white squares) in host cell mitochondria, cytosol, nucleus and chlamydial inclusion at 24 hpi (n = 3; mean ±SEM). Fluorescence intensity values were normalized to the fluorescence intensity of the media in each emission channels. There is no shift in the peak emission wavelengths of autofluorescence originating from the chlamydial inclusion compared to host cell mitochondria. The peak of emission between 450–500 nm at 730 nm excitation by two-photon microscopy corresponds to the peak of the NAD(P)H fluorescence spectrum.
(TIF)

Figure S2 **Autofluorescence intensity and frequency distribution of τ_2_-NAD(P)H.** (A) Autofluorescence intensities were used to define different cellular compartments (upper panels) and ROIs were transferred to color-coded images of τ_2_-NAD(P)H (lower panels) of a non-infected and a *C. trachomatis*-infected cell at 24 hpi (scale bar =  10 µm; M = mitochondria, I = inclusion, N = nucleus). The line in the *C. trachomatis*-infected cell (upper right) marks the area that was used for analysis of fluorescence intensity in Figure S2B. (B) Analysis of the fluorescence intensity of the line shown in Figure S2A in *C. trachomatis*-infected cell. The fluorescence intensity is different in host cell mitochondria, nucleus and inside the chlamydial inclusion indicating the different concentration of NAD(P)H in these cellular compartments. The different intensity signal enables the separation of these cellular compartments (M = mitochondria, I = inclusion, N = nucleus). (C) Histogram of τ_2_-NAD(P)H in the cytosolic, mitochondrial and nuclear compartments of non-infected cells and in the chlamydial inclusion of infected cells at 24 hpi (n = 54 from three independent experiments).
(TIF)

Figure S3 **Co-localization analysis of NAD(P)H fluorescence with the mitochondria marker, TMRE.** (A) Intensity profile (middle panel) of the line indicated in the overlay picture (upper panel) and in the magnified picture (lower panel) show complete co-localization of TMRE and NAD(P)H fluorescence in non-infected HEp-2 cells. (B) Intensity profile (middle panel) of the line indicated in the overlay picture (upper panel) and in the magnified picture (lower panel) show co-localization of TMRE and NAD(P)H fluorescence in the mitochondria but not in the chlamydial inclusion and on the inclusion membrane in *C. trachomatis*-infected HEp-2 cell at 24 hpi.
(TIF)

Figure S4 **FLIM analysis of** ***C. trachomatis*** **metabolism.** (A) The scatter plot shows the homogenous distribution of the average τ_2_-NAD(P)H values in *C. trachomatis* inclusions at 24 hpi and the heterogeneous distribution at 48 hpi (n = 54 from three independent experiments). (B) Quantitative analysis of the ratio of free to protein-bound NAD(P)H (a_1_/a_2_) inside the *C. trachomatis* inclusion of NAD(P)H FLIM images after the indicated time points of infection (n = 54; mean±SEM). Detailed results of statistical analysis are shown in Table S3B.
(TIF)

Figure S5 **NAD(P)H autofluorescence intensity in the chlamydial inclusion 18 hpi, 24 hpi and 48 hpi.** (A) Pseudo-colour images of NAD(P)H fluorescence intensity measured by FLIM in *C. trachomatis*-infected cells 18 hpi, 24 hpi and 48 hpi (scale bars = 20 µm). (B) Histogram of NAD(P)H autofluorescence distribution inside the chlamydial inclusion after 18 hours (n = 442 pixels), 24 hours (n = 1060 pixels) and 48 hours (n = 1561 pixels) of infection. (C) Enlarged images of representative cells used for NAD(P)H fluorescence intensity analysis (scale bars = 10 µm; I = inclusion, N = nucleus).
(TIF)

Figure S6 **Changes of τ_2_-NAD(P)H inside** ***C. pneumoniae*** **inclusions during the intracellular developmental cycle.** (A) Changes of τ_2_-NAD(P)H inside the *C. pneumoniae* inclusion during the bacterial developmental cycle. HEp-2 cells were infected with *C. pneumoniae* for 36, 48, 60, 72, 84 and 96 hours. (B) Quantitative analysis of τ_2_-NAD(P)H inside the *C. pneumoniae* inclusion and of *C. pneumoniae* inclusion sizes (n = 36 (36 hpi); n = 32 (48 hpi); n = 42 (60 hpi); n = 27 (84 hpi); n = 18 (96 hpi) from four independent experiments; mean±SEM).
(TIF)

Figure S7 **Impact of host-cell metabolism inhibition on ATP levels and τ_2_-NAD(P)H.** (A) Cellular ATP levels under glucose starvation and inhibition of oxidative phosphorylation by antimycin A. (B) Histogram of τ_2_-NAD(P)H in the *C.trachomatis*-inclusions under different metabolic perturbations.
(TIF)

Table S1 **Quantitative analysis of NAD(P)H FLIM.** Fluorescence lifetimes, relative amounts and fluorescence quantum yields of free and protein-bound NAD(P)H in the cytosol, mitochondria and nucleus of non-infected HEp-2 cells and in the *C. trachomatis* inclusion. τ_1_: fluorescence lifetime of free NAD(P)H, τ_2_: fluorescence lifetime of protein-bound NAD(P)H, a_1_: relative amount of free NAD(P)H, a_2_: relative amount of protein-bound NAD(P)H, q_1_: fluorescence quantum yield of free NAD(P)H, q_2_: fluorescence quantum yield of protein-bound NAD(P)H (n = 54; mean ± SD).
(DOC)

Table S2 **Statistical analysis of τ_2_-NAD(P)H in cytosol, mitochondria, nucleus of non-infected HEp-2 and in chlamydial inclusion at 24 hpi.** The model included experimental days (three per group, hence six in total) and compartment (cytosol, mitochondria, nucleus, inclusion) as independent factors and images per day (six) as well as cells per image (three) as repeated measures with all main effected and interactions. The dependent variable was τ_2_-NAD(P)H in nucleus. Compartment was tested overall as well as comparing inclusion with every other compartment.
(DOC)

Table S3 **Statistical analysis of τ_2_-NAD(P)H and a_1_/a_2_ in the chlamydial inclusion 12 hpi, 24 hpi and 48 hpi.** The model included experimental days (three per group, hence six in total) and time point (comparison 1: 12hpi vs 24hpi, comparison 2: 24hpi vs 48hpi) as independent factors and images per day (six) as well as cells per image (three) as repeated measures with all main effected and interactions. The dependent variable were τ_2_-NAD(P)H (A) and a_1_/a_2_ (B). Differences in time points were tested.
(DOC)

Table S4 **Statistical analysis of τ_2_-NAD(P)H in the chlamydial inclusion under different metabolic inhibition conditions at 24 hpi.** The model included experimental days (three per group, hence six in total) and treatment (comparison 1: control vs no glucose, comparison 2: control vs antimycin A) as independent factors and images per day (six) as well as cells per image (three) as repeated measures with all main effected and interactions. The dependent variable was τ_2_-NAD(P)H.
(DOC)

Table S5 **Statistical analysis of τ_2_-NAD(P)H and a_1_/a_2_ in the host cell nucleus.** The models included experimental days (three per group, hence six in total) and treatment (comparison 1: 2FDG vs controls, comparison 2: *C. trachomatis* at 24 hpi vs controls) as independent factors and images per day (six) as well as cells per image (three) as repeated measures with all main effected and interactions. The dependent variables were τ_2_-NAD(P)H (A) and a_1_/a_2_ (B).
(DOC)
