# Supplementary figures and images for: Fluorescence Lifetime Imaging Unravels C. trachomatis Metabolism and Its Crosstalk with the Host Cell
Source: PLoS Pathog. 2011 Jul 14;7(7):e1002108. doi: 10.1371/journal.ppat.1002108 (PMC3136453; doi:10.1371/journal.ppat.1002108)

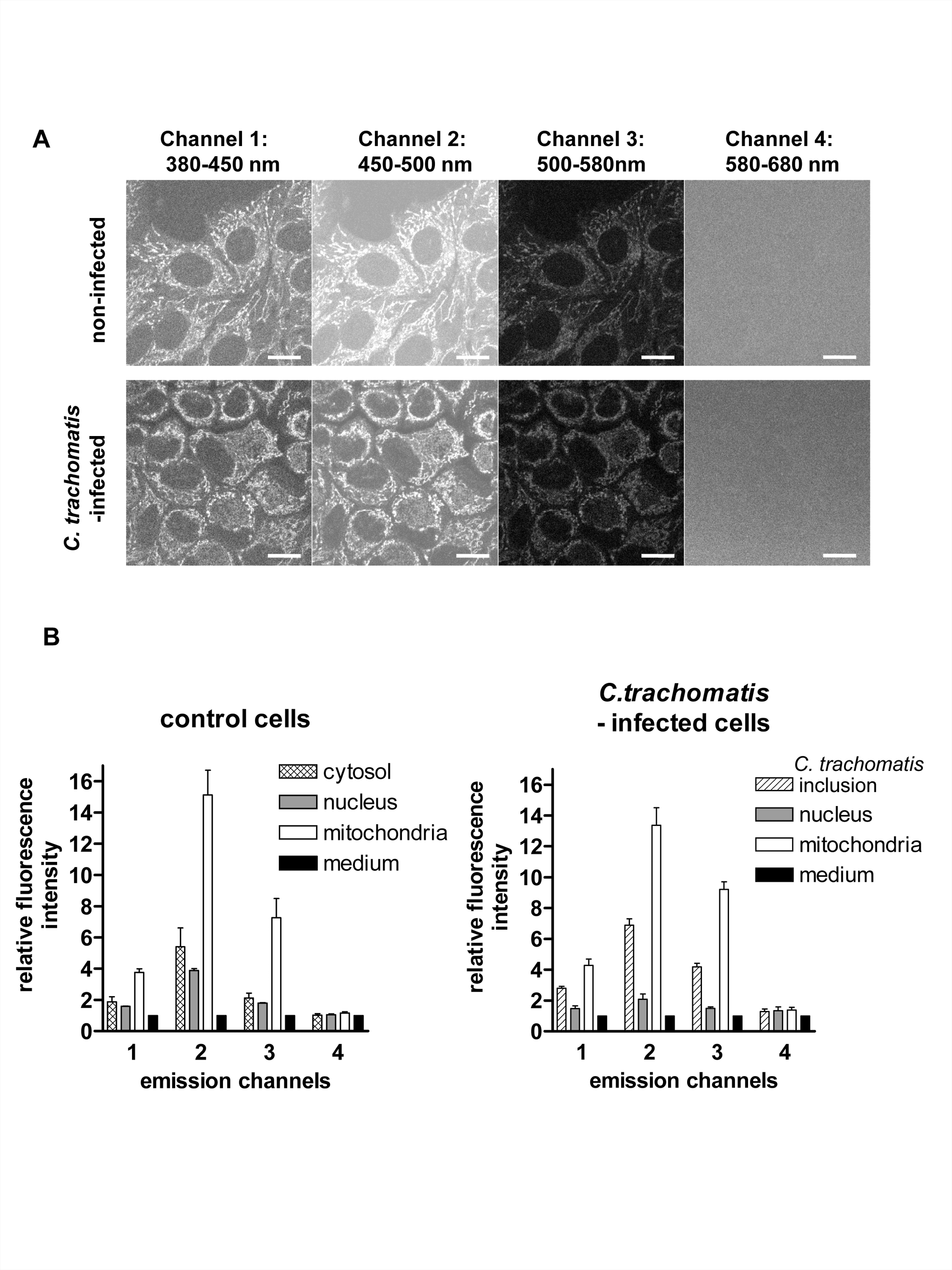

Supplement: Figure S1 — Spectral characterization of autofluorescence signals at 730 nm excitation. (A) Spectral characterization of autofluorescence signals of non-infected HEp-2 cells (upper panels) and of C. trachomatis-infected cells at 24 hpi (lower panels) shows typical NAD(P)H fluorescence (scale bar = 20 µm). (B) Quantification of fluorescence intensity of selected ROIs (white squares) in host cell mitochondria, cytosol, nucleus and chlamydial inclusion at 24 hpi (n = 3; mean ±SEM). Fluorescence intensity values were normalized to the fluorescence intensity of the media in each emission channels. There is no shift in the peak emission wavelengths of autofluorescence originating from the chlamydial inclusion compared to host cell mitochondria. The peak of emission between 450–500 nm at 730 nm excitation by two-photon microscopy corresponds to the peak of the NAD(P)H fluorescence spectrum. (TIF) [file ppat.1002108.s001.tif]

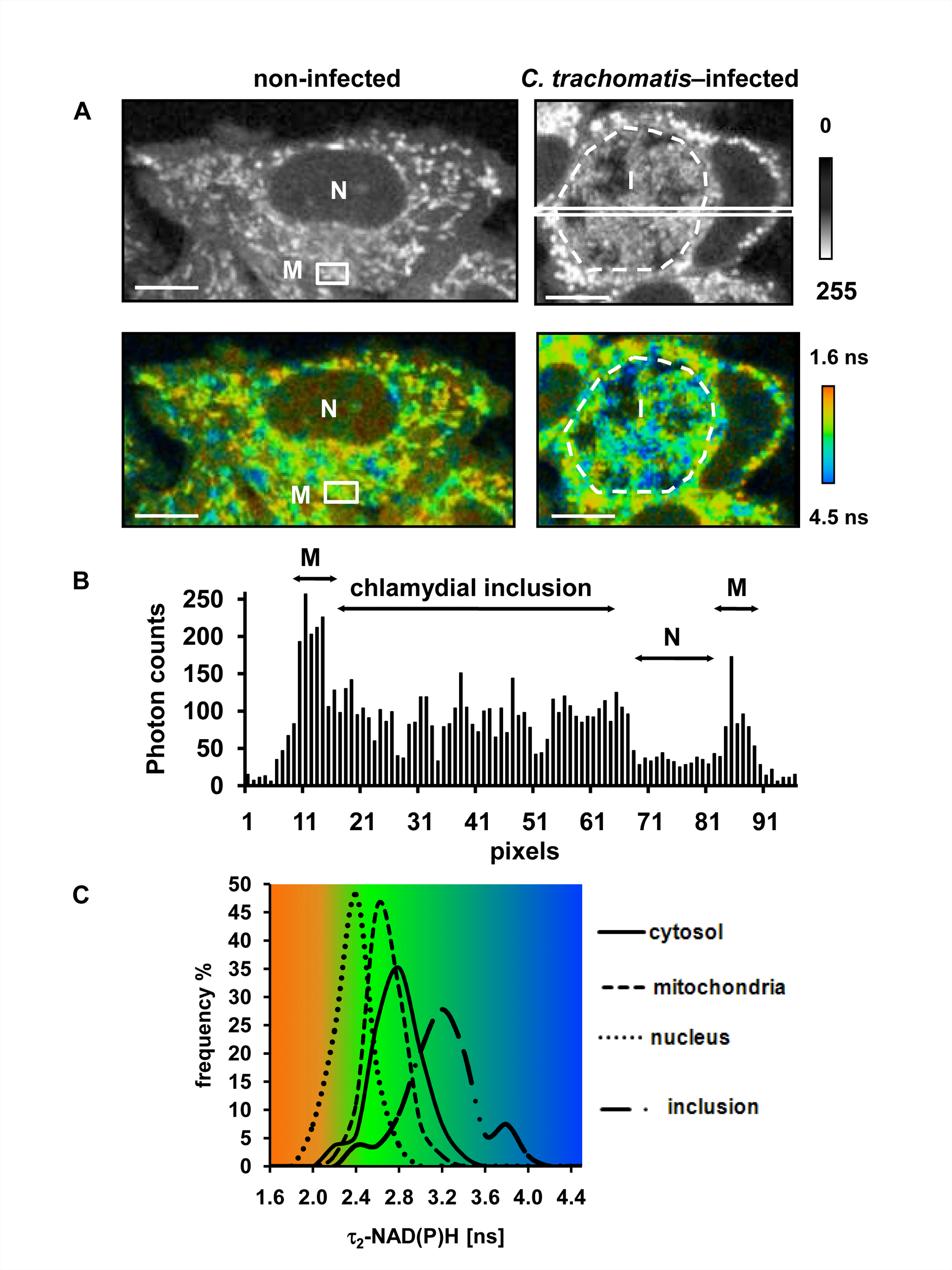

Supplement: Figure S2 — Autofluorescence intensity and frequency distribution of τ2-NAD(P)H. (A) Autofluorescence intensities were used to define different cellular compartments (upper panels) and ROIs were transferred to color-coded images of τ2-NAD(P)H (lower panels) of a non-infected and a C. trachomatis-infected cell at 24 hpi (scale bar = 10 µm; M = mitochondria, I = inclusion, N = nucleus). The line in the C. trachomatis-infected cell (upper right) marks the area that was used for analysis of fluorescence intensity in Figure S2B. (B) Analysis of the fluorescence intensity of the line shown in Figure S2A in C. trachomatis-infected cell. The fluorescence intensity is different in host cell mitochondria, nucleus and inside the chlamydial inclusion indicating the different concentration of NAD(P)H in these cellular compartments. The different intensity signal enables the separation of these cellular compartments (M = mitochondria, I = inclusion, N = nucleus). (C) Histogram of τ2-NAD(P)H in the cytosolic, mitochondrial and nuclear compartments of non-infected cells and in the chlamydial inclusion of infected cells at 24 hpi (n = 54 from three independent experiments). (TIF) [file ppat.1002108.s002.tif]

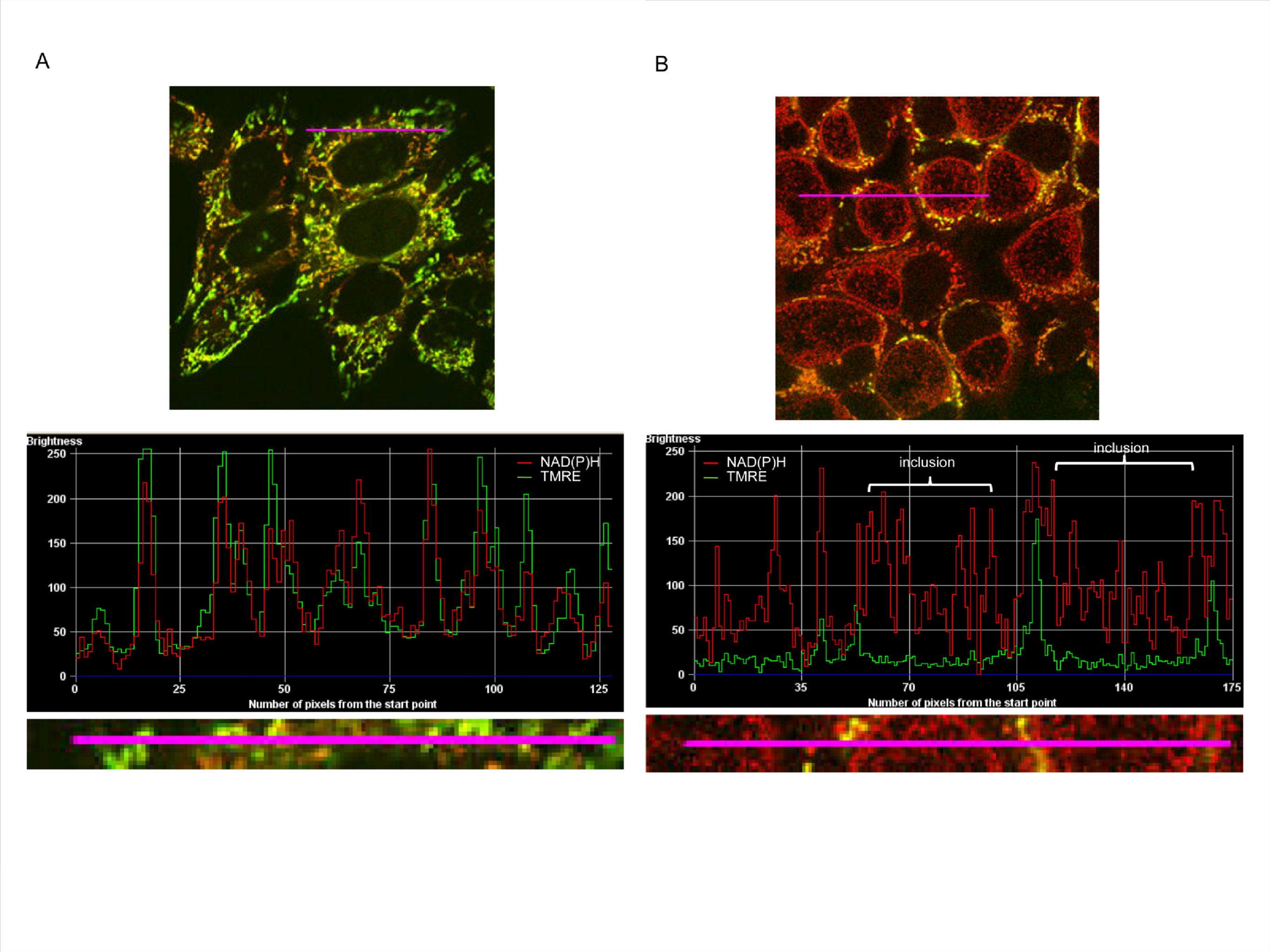

Supplement: Figure S3 — Co-localization analysis of NAD(P)H fluorescence with the mitochondria marker, TMRE. (A) Intensity profile (middle panel) of the line indicated in the overlay picture (upper panel) and in the magnified picture (lower panel) show complete co-localization of TMRE and NAD(P)H fluorescence in non-infected HEp-2 cells. (B) Intensity profile (middle panel) of the line indicated in the overlay picture (upper panel) and in the magnified picture (lower panel) show co-localization of TMRE and NAD(P)H fluorescence in the mitochondria but not in the chlamydial inclusion and on the inclusion membrane in C. trachomatis-infected HEp-2 cell at 24 hpi. (TIF) [file ppat.1002108.s003.tif]

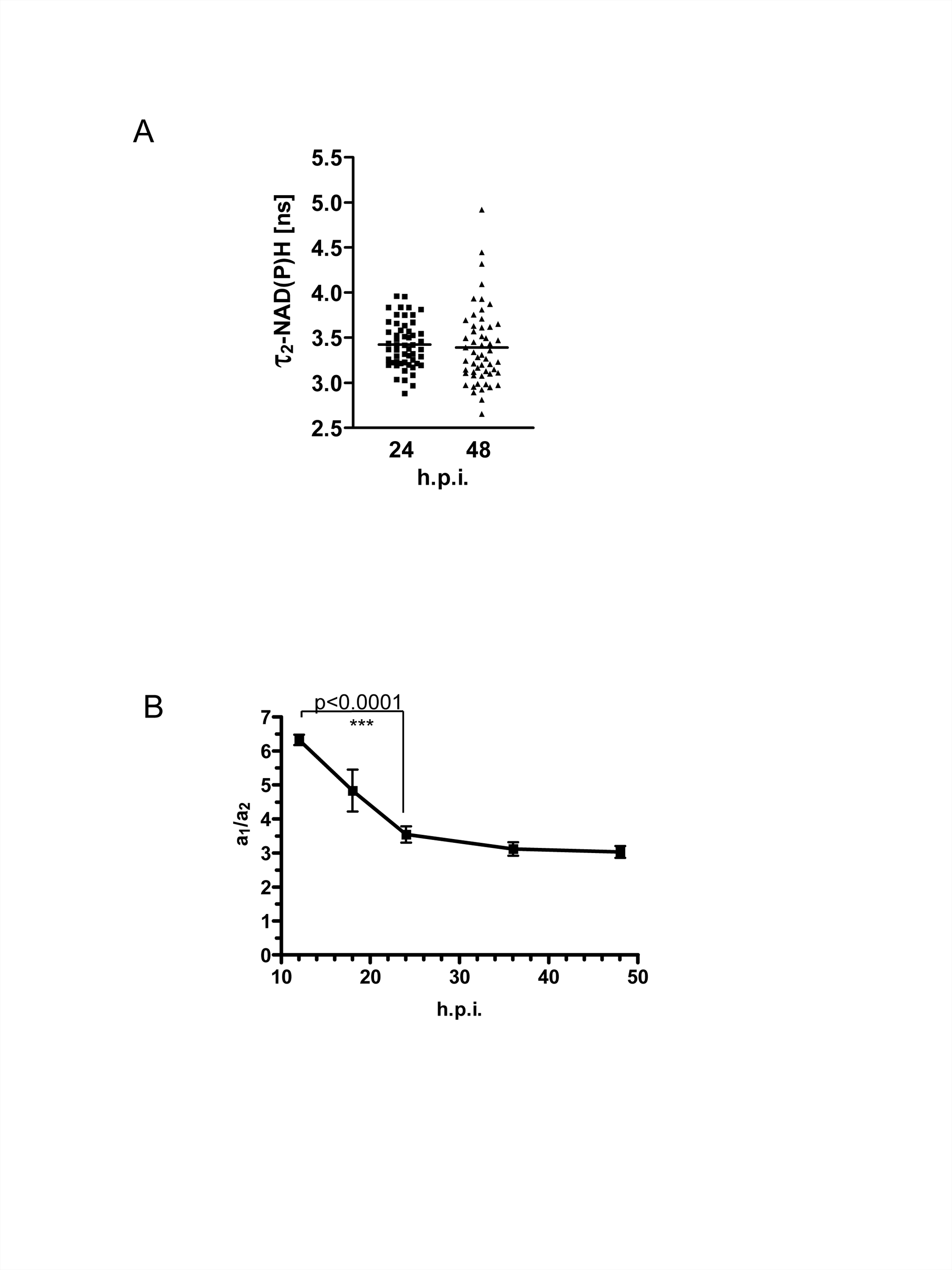

Supplement: Figure S4 — FLIM analysis of C. trachomatis metabolism. (A) The scatter plot shows the homogenous distribution of the average τ2-NAD(P)H values in C. trachomatis inclusions at 24 hpi and the heterogeneous distribution at 48 hpi (n = 54 from three independent experiments). (B) Quantitative analysis of the ratio of free to protein-bound NAD(P)H (a1/a2) inside the C. trachomatis inclusion of NAD(P)H FLIM images after the indicated time points of infection (n = 54; mean±SEM). Detailed results of statistical analysis are shown in Table S3B. (TIF) [file ppat.1002108.s004.tif]

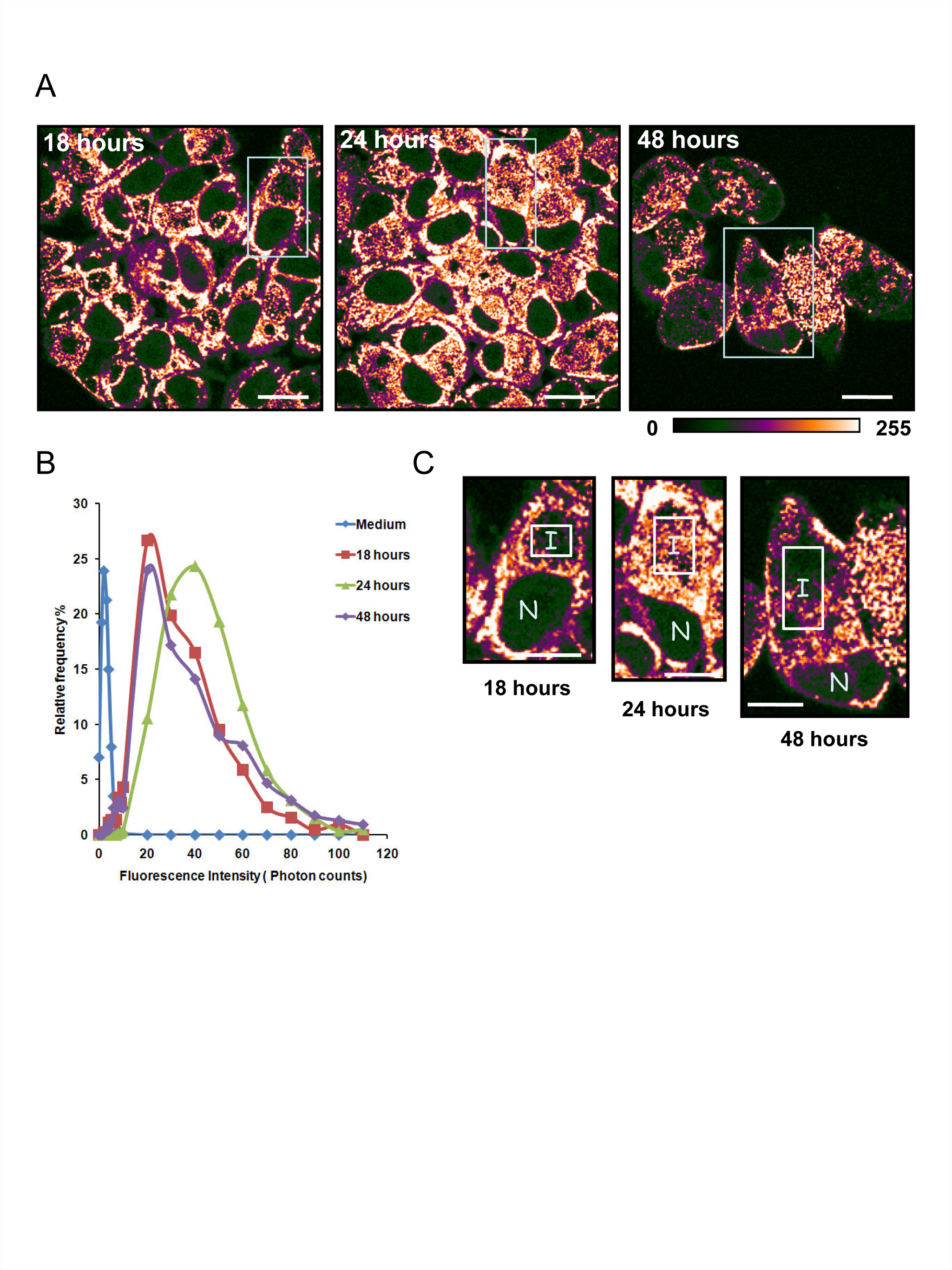

Supplement: Figure S5 — NAD(P)H autofluorescence intensity in the chlamydial inclusion 18 hpi, 24 hpi and 48 hpi. (A) Pseudo-colour images of NAD(P)H fluorescence intensity measured by FLIM in C. trachomatis-infected cells 18 hpi, 24 hpi and 48 hpi (scale bars = 20 µm). (B) Histogram of NAD(P)H autofluorescence distribution inside the chlamydial inclusion after 18 hours (n = 442 pixels), 24 hours (n = 1060 pixels) and 48 hours (n = 1561 pixels) of infection. (C) Enlarged images of representative cells used for NAD(P)H fluorescence intensity analysis (scale bars = 10 µm; I = inclusion, N = nucleus). (TIF) [file ppat.1002108.s005.tif]

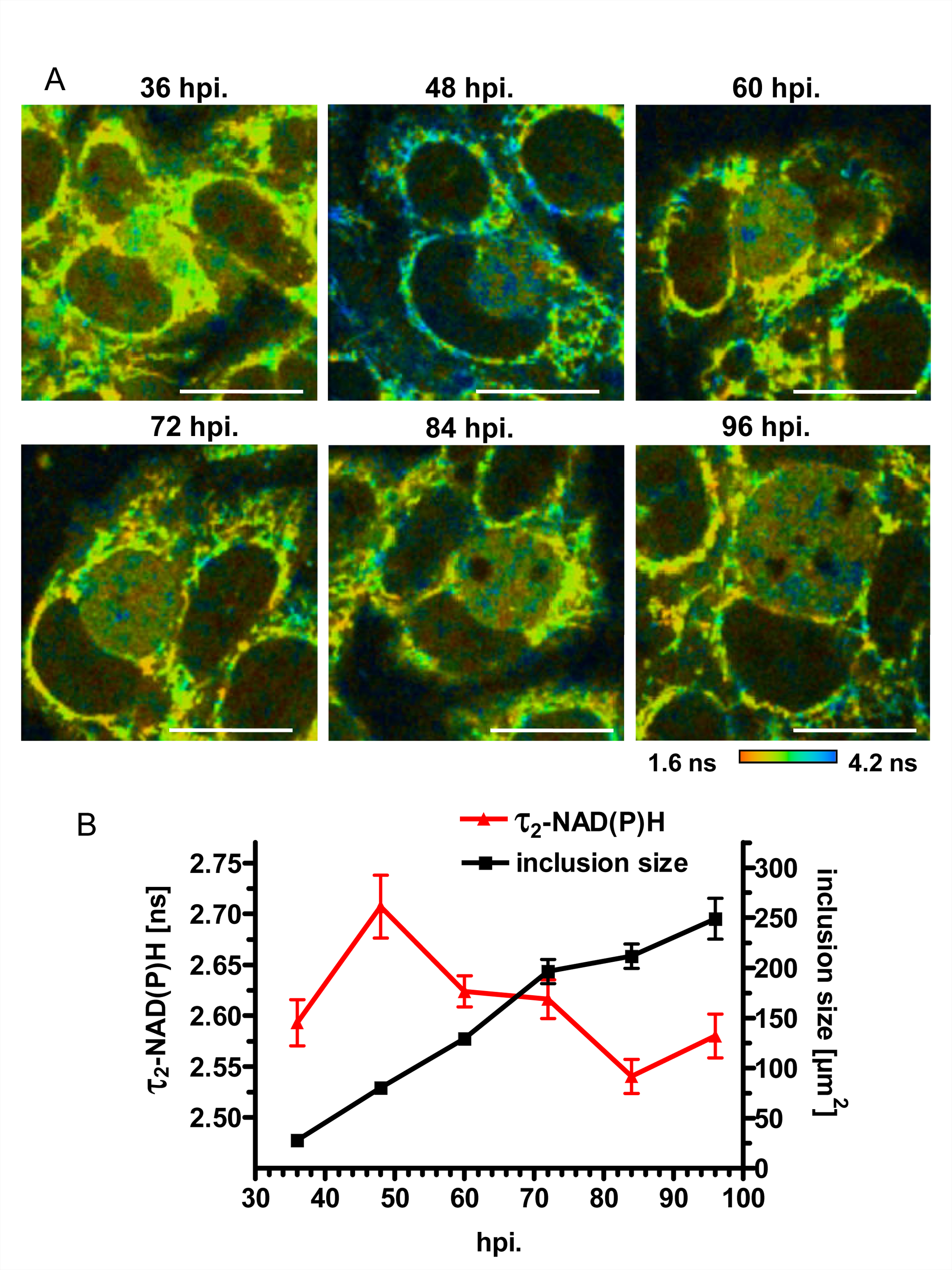

Supplement: Figure S6 — Changes of τ2-NAD(P)H inside C. pneumoniae inclusions during the intracellular developmental cycle. (A) Changes of τ2-NAD(P)H inside the C. pneumoniae inclusion during the bacterial developmental cycle. HEp-2 cells were infected with C. pneumoniae for 36, 48, 60, 72, 84 and 96 hours. (B) Quantitative analysis of τ2-NAD(P)H inside the C. pneumoniae inclusion and of C. pneumoniae inclusion sizes (n = 36 (36 hpi); n = 32 (48 hpi); n = 42 (60 hpi); n = 27 (84 hpi); n = 18 (96 hpi) from four independent experiments; mean±SEM). (TIF) [file ppat.1002108.s006.tif]

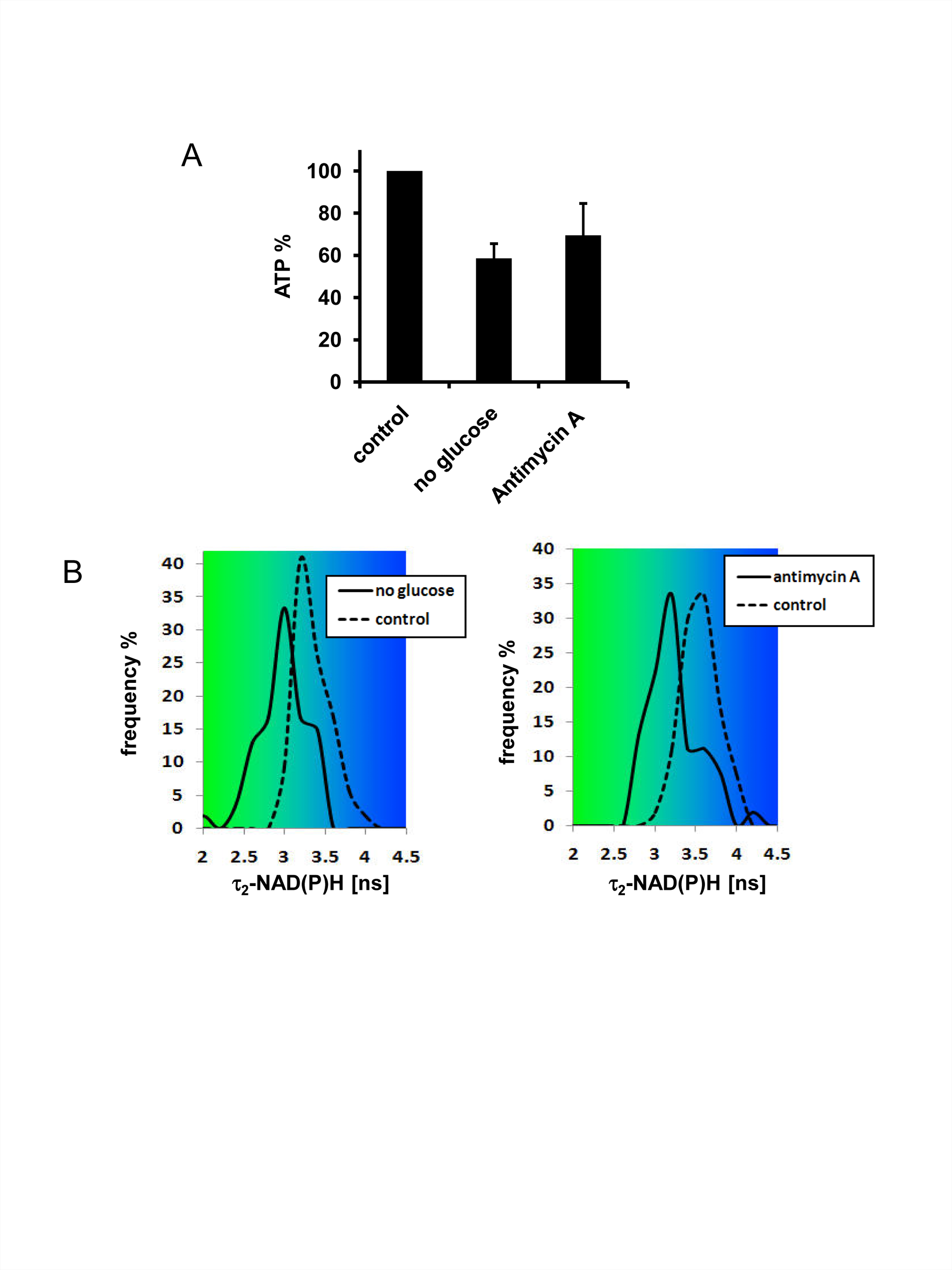

Supplement: Figure S7 — Impact of host-cell metabolism inhibition on ATP levels and τ2-NAD(P)H. (A) Cellular ATP levels under glucose starvation and inhibition of oxidative phosphorylation by antimycin A. (B) Histogram of τ2-NAD(P)H in the C.trachomatis-inclusions under different metabolic perturbations. (TIF) [file ppat.1002108.s007.tif]
