# Supplementary material for: Fluorescence Lifetime Imaging Unravels C. trachomatis Metabolism and Its Crosstalk with the Host Cell
Source: PLoS Pathog. 2011 Jul 14;7(7):e1002108. doi: 10.1371/journal.ppat.1002108 (PMC3136453; doi:10.1371/journal.ppat.1002108)
Supplement: Table S1 — Quantitative analysis of NAD(P)H FLIM. Fluorescence lifetimes, relative amounts and fluorescence quantum yields of free and protein-bound NAD(P)H in the cytosol, mitochondria and nucleus of non-infected HEp-2 cells and in the C. trachomatis inclusion. τ1: fluorescence lifetime of free NAD(P)H, τ2: fluorescence lifetime of protein-bound NAD(P)H, a1: relative amount of free NAD(P)H, a2: relative amount of protein-bound NAD(P)H, q1: fluorescence quantum yield of free NAD(P)H, q2: fluorescence quantum yield of protein-bound NAD(P)H (n = 54; mean ± SD). (DOC) [file ppat.1002108.s008.doc]

|  | **τ_1_ [ns]** | **τ_2_ [ns]** | **a_1_ [%]** | **a_2_[%]** | **q_1_[%]** | **q_2_[%]** |
| --- | --- | --- | --- | --- | --- | --- |
| **cytosol** | 0.49 ± 0.11 | 2.69 ± 0.24 | 79.10 ± 1.62 | 20.90 ± 1.62 | 40.35 ± 3.53 | 59.65 ± 3.53 |
| **mitochondria** | 0.38 ± 0.06 | 2.57 ± 0.17 | 81.86 ± 2.07 | 18.14 ± 2.07 | 40.09 ± 3.24 | 59.91 ± 3.24 |
| **nucleus** | 0.43 ± 0.07 | 2.27 ± 0.17 | 83.63 ± 1.70 | 16.37 ± 1.70 | 49.65 ± 2.74 | 50.35 ± 2.74 |
| **inclusion** | 0.46 ± 0.12 | 3.10 ± 0.34 | 80.47 ± 3.96 | 19.53 ± 3.96 | 38.47 ± 6.02 | 61.54 ± 6.02 |

**Table S1**
