# Supplementary material for: Fluorescence Lifetime Imaging Unravels C. trachomatis Metabolism and Its Crosstalk with the Host Cell
Source: PLoS Pathog. 2011 Jul 14;7(7):e1002108. doi: 10.1371/journal.ppat.1002108 (PMC3136453; doi:10.1371/journal.ppat.1002108)
Supplement: Table S2 — Statistical analysis of τ2-NAD(P)H in cytosol, mitochondria, nucleus of non-infected HEp-2 and in chlamydial inclusion at 24 hpi. The model included experimental days (three per group, hence six in total) and compartment (cytosol, mitochondria, nucleus, inclusion) as independent factors and images per day (six) as well as cells per image (three) as repeated measures with all main effected and interactions. The dependent variable was τ2-NAD(P)H in nucleus. Compartment was tested overall as well as comparing inclusion with every other compartment. (DOC) [file ppat.1002108.s009.doc]

**Table S2**

| **τ_2_-NAD(P)H** | **nominal p** | **Bonf-Holm** | **Bonferroni** |
| --- | --- | --- | --- |
|  |  | **set-wise** | **overall** |
| **inclusion vs. cytosol** | 0.02054 | 0.02054 | 0.16432 |
| **inclusion vs. nucleus** | 0.0001 | 0.0003 | 0.0024 |
| **inclusion vs. mitochondria** | 0.00021 | 0.00042 | 0.00336 |
