# Supplementary material for: Fluorescence Lifetime Imaging Unravels C. trachomatis Metabolism and Its Crosstalk with the Host Cell
Source: PLoS Pathog. 2011 Jul 14;7(7):e1002108. doi: 10.1371/journal.ppat.1002108 (PMC3136453; doi:10.1371/journal.ppat.1002108)
Supplement: Table S3 — Statistical analysis of τ2-NAD(P)H and a1/a2 in the chlamydial inclusion 12 hpi, 24 hpi and 48 hpi. The model included experimental days (three per group, hence six in total) and time point (comparison 1: 12hpi vs 24hpi, comparison 2: 24hpi vs 48hpi) as independent factors and images per day (six) as well as cells per image (three) as repeated measures with all main effected and interactions. The dependent variable were τ2-NAD(P)H (A) and a1/a2 (B). Differences in time points were tested. (DOC) [file ppat.1002108.s010.doc]

**Table S3**

A

| **τ_2_-NAD(P)H** | **nominal p** | **Bonf-Holm** | **Bonferroni** |
| --- | --- | --- | --- |
|  |  | **set-wise** | **overall** |
| **12h vs. 24h** | 0.0001 | 0.0002 | 0.0016 |
| **24h vs. 48h** | 0.62844 | 0.62844 | 1 |

B

| **a_1_/a_2_** | **nominal p** | **Bonf-Holm** | **Bonferroni** |
| --- | --- | --- | --- |
|  |  | **set-wise** | **overall** |
| **12h vs. 24h** | 0.0001 | 0.0002 | 0.0016 |
| **24h vs. 48h** | 0.85809 | 0.85809 | 1 |
