# Supplementary material for: Fluorescence Lifetime Imaging Unravels C. trachomatis Metabolism and Its Crosstalk with the Host Cell
Source: PLoS Pathog. 2011 Jul 14;7(7):e1002108. doi: 10.1371/journal.ppat.1002108 (PMC3136453; doi:10.1371/journal.ppat.1002108)
Supplement: Table S4 — Statistical analysis of τ2-NAD(P)H in the chlamydial inclusion under different metabolic inhibition conditions at 24 hpi. The model included experimental days (three per group, hence six in total) and treatment (comparison 1: control vs no glucose, comparison 2: control vs antimycin A) as independent factors and images per day (six) as well as cells per image (three) as repeated measures with all main effected and interactions. The dependent variable was τ2-NAD(P)H. (DOC) [file ppat.1002108.s011.doc]

**Table S4**

| **τ_2_-NAD(P)H** | **nominal p** | **Bonf-Holm** | **Bonferroni** |
| --- | --- | --- | --- |
|  |  | **set-wise** | **overall** |
| **control vs. no glucose** | 0.00016 | 0.00016 | 0.00128 |
| **control vs. antimycin A** | 0.0001 | 0.0002 | 0.0016 |
