# Supplementary material for: Fluorescence Lifetime Imaging Unravels C. trachomatis Metabolism and Its Crosstalk with the Host Cell
Source: PLoS Pathog. 2011 Jul 14;7(7):e1002108. doi: 10.1371/journal.ppat.1002108 (PMC3136453; doi:10.1371/journal.ppat.1002108)
Supplement: Table S5 — Statistical analysis of τ2-NAD(P)H and a1/a2 in the host cell nucleus. The models included experimental days (three per group, hence six in total) and treatment (comparison 1: 2FDG vs controls, comparison 2: C. trachomatis at 24 hpi vs controls) as independent factors and images per day (six) as well as cells per image (three) as repeated measures with all main effected and interactions. The dependent variables were τ2-NAD(P)H (A) and a1/a2 (B). (DOC) [file ppat.1002108.s012.doc]

**Table S5**

A

| **τ_2_-NAD(P)H** | **nominal p** | **Bonf-Holm** | **Bonferroni** |
| --- | --- | --- | --- |
|  |  | **set-wise** | **overall** |
| **non-infected vs. +2FDG** | 0.0001 | 0.0002 | 0.0016 |
| **non-infected vs. +*C.trachomatis*** | 0.00189 | 0.00189 | 0.01512 |

B

| **a_1_/a_2_** | **nominal p** | **Bonf-Holm** | **Bonferroni** |
| --- | --- | --- | --- |
|  |  | **set-wise** | **overall** |
| **non-infected vs. +2FDG** | 0.0001 | 0.0002 | 0.0016 |
| **non-infected vs. +*C.trachomatis*** | 0.00108 | 0.00108 | 0.00864 |
